# Unraveling the herpetofauna diversity in *canga* and forest ecosystems of the Eastern Amazon

**DOI:** 10.1371/journal.pone.0332753

**Published:** 2025-11-26

**Authors:** Ana Lúcia Costa Prudente, Fernanda Magalhães Silva, Marina Meireles dos Santos, Santelmo Vasconcelos, Fernando J. M. Rojas-Runjaic, Adriana C. Becerra-Rondón, Adriano Oliveira Maciel, João Fabrício Sarmento, Roberta Graboski, Cássia Teixeira, Alessandra C. Guimarães, Aline Nascimento, Amanda M. S. Oliveira, Michele Molina, Patricia Silva, Cesar de Sá Carvalho Neto, Gisele Lopes Nunes

**Affiliations:** 1 Coordenação de Zoologia, Museu Paraense Emílio Goeldi, Belém, Pará, Brazil; 2 Programa de Pós-Graduação em Biodiversidade e Evolução, Museu Paraense Emílio Goeldi, Belém, Pará, Brazil; 3 Programa de Pós-Graduação em Zoologia, Universidade Federal do Pará e Museu Paraense Emílio Goeldi, Belém, Pará, Brazil; 4 Instituto Tecnológico Vale, Belém, Pará, Brazil; 5 Museo de Historia Natural La Salle, Fundación La Salle de Ciencias Naturales, Caracas, Capital District, Venezuela; 6 Vale S.A, Parauapebas, Pará, Brazil; University of Rome, ITALY

## Abstract

Species inventories are essential for discovering new taxa, improving knowledge of species’ geographic distributions, characterizing local richness, evaluating biodiversity loss, and contributing to the conservation of endangered areas, including those with endemic and rare species. The southeastern region of Pará, Brazil, encompasses a transitional zone between the Amazon and Cerrado biomes, marked by a mosaic of natural environments with high variability in relief, substrates, and geological attributes. We conducted a comprehensive survey of the region’s herpetofauna, combining taxonomic surveys with molecular characterization, with a particular focus on species associated with savanna-like environments known as *canga*. We selected four sampling sites: one within the Serra dos Carajás mosaic of protected areas and three in the surrounding region, including São Geraldo do Araguaia, Conceição do Araguaia, and Ourilândia do Norte/São Félix do Xingu. Our inventory recorded a total of 242 species (99 amphibians and 143 squamate reptiles), including ten new records for the state of Pará and two notable range extensions. We also generated a DNA barcode reference library of 860 sequences (436 COI and 424 16S rRNA) from 500 specimens. Approximately 58.4% of amphibian species and 32.2% of squamate reptile species were supported by at least one reference barcode. Our dataset includes five novel COI and two novel 16S rRNA records for amphibians, and 25 novel COI and 13 novel 16S rRNA records for squamate reptiles.

## Introduction

The Neotropical region harbors exceptionally high levels of biodiversity and endemism, primarily due to its complex topography, heterogeneous ecoregions, and wide range of habitats, including the Amazon rainforest, one of the most biodiverse ecosystems on Earth [1–5]. Biodiversity inventories play a critical role in addressing the Wallacean shortfall in megadiverse regions. These studies are crucial not only for discovering new species and mapping distribution patterns but also for advancing knowledge of species ranges and generating both ecological and molecular datasets. In addition, they contribute significantly to the enhancement of scientific collections, which are vital resources for taxonomic, ecological, and conservation research [6,7].

Data generated from biological inventories directly inform assessments of species’ conservation status and support the prioritization of regions or taxa for management. This represents a significant challenge, as effective conservation efforts rely on a comprehensive understanding of the region [3,8]. Consequently, systematic and taxonomic studies are essential for accurately characterizing species richness, interpreting diversity patterns, and understanding mechanisms of diversification. However, species-level identification can be challenged by cryptic diversity and morphological similarity, as well as a substantial amount of undescribed species [8,9]. Advancements in molecular methods, particularly DNA sequencing, have greatly enhanced taxonomic and biodiversity studies, especially with the development of a technique known as DNA barcoding [9–11]. Over the past decade, DNA-based identification using DNA barcoding has become widely employed in documenting herpetofauna, resolving taxonomic ambiguities and detecting hidden diversity (e.g., [11–18]).

DNA barcoding facilitates the identification of undescribed or closely related species, even in cases involving juvenile organisms without discernible traits, shed skins, bones, eggs, larvae, or species characterized by subtle morphological variation [10,19]. This technique also supports phylogeographic analyses by enabling comparisons of genetic lineages. It is increasingly applied in environmental DNA (eDNA) studies through DNA metabarcoding, enhancing environmental monitoring studies [20,21]. Both methodologies typically employ mitochondrial DNA (mtDNA) due to its utility in assessing intra- and interspecific variation. The mitochondrial genome is a circular, double-stranded DNA (dsDNA) molecule (~ 16,500 bp) containing 37 genes, 13 related to respiratory chain function and 24 encoding components of the mitochondrial translation system, including tRNAs and rRNAs. Among these, the cytochrome c oxidase subunit I (COI) gene, a ~ 650 bp fragment, is considered a universal genetic marker for animal identification due to the accumulation of specific mutations that allows species differentiation [10]. In addition to COI, other mtDNA markers such as cytochrome b (Cytb), ribosomal 12S rRNA and 16S rRNA are also commonly used for their hypervariable regions, which enable population-level resolution and species discrimination (e.g., [12,13,19,20]).

Among the world’s megadiverse regions, Brazil stands out for its vast territory in South America. The Amazon biome, 65% of which lies within Brazil, is among the most studied and ecologically significant ecosystems on the planet [1,3]. Despite this, large areas of the Brazilian Amazon remain underexplored, particularly in terms of their herpetofauna [1,22–25]. In the easternmost portion of the Amazon, specifically in southeastern Pará state, complex landscapes with rich geodiversity are present, featuring ironstone rocky outcrop formations known as *Campos Rupestres* or *canga*, located on extensive plateaus in the Serra dos Carajás. These ferruginous savanna-like ecosystems represent unique landscapes within the dense forest matrix typical of the Amazon rainforest and are recognized for their remarkable diversity and high levels of endemism [26–28]. Since the 1970s, southeastern Pará has undergone extensive land-use change, with vast forest areas converted to pasture and other anthropogenic uses [29]. This transformation poses severe threats to species with restricted distributions, including those already at risk [30,31].

The herpetofauna of southeastern Pará primarily consists of species typically found in the Amazon biome, although some have broader distributions extending into the Cerrado biome. Notable examples include the anurans *Leptodactylus syphax* and *Pseudis tocantins* [32], and the lizards *Norops brasiliensis* and *Notomabuya frenata* [33]. One of the most extensively studied areas for its fauna and flora in the region is the *Floresta Nacional de Carajás* (Carajás National Forest, CNF), a mosaic of natural environments with enclaves of Cerrado vegetation, surrounded by anthropized areas. This forest, along with five other protected areas, forms the Carajás mosaic of conservation units, an area of approximately 13,000 km² that holds significant importance for the biological conservation of the Amazon [34,35].

In this study, we conducted a comprehensive survey of amphibians and squamate reptiles in southeastern Pará, focusing on the *canga* formations and associated forest environments, aiming to address and mitigate the Wallacean shortfall. Our objectives were to: (i) compile a comprehensive species inventory and assess taxonomic richness; (ii) establish a DNA barcoding reference database alongside a specimen collection; and (iii) evaluate the performance of COI and 16S rRNA markers for species differentiation in amphibians and squamate reptiles. The resulting data improve our understanding of local species richness, geographic distribution, and diversity patterns, which are critical for assessing conservation priorities within this biologically important region.

## Materials and methods

### Study area

**Fig 1:**
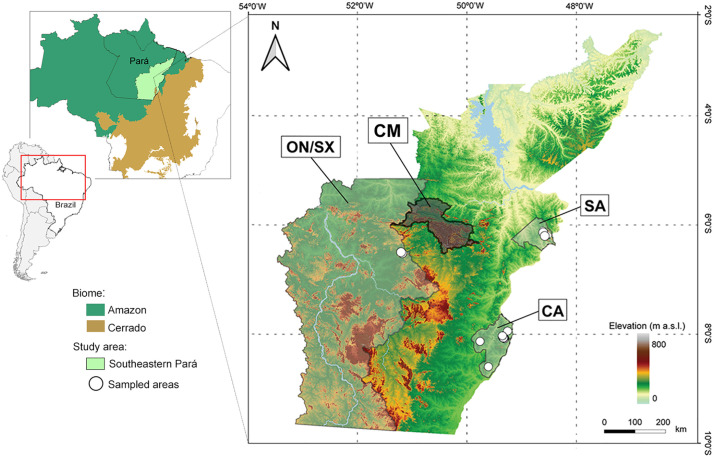
Map displaying the study area in southeastern Pará, Brazil, Eastern Amazon. Carajás Mosaic (CM), Conceição do Araguaia (CA), São Geraldo do Araguaia (SA), and Ourilândia do Norte/São Félix do Xingu (ON/SX). Map created by the authors using public domain data from Natural Earth and IBGE (www.naturalearthdata.com; www.ibge.gov.br).

The study area encompasses the mosaic of protected areas located in the Serra dos Carajás, hereafter referred to as the Carajás Mosaic (CM), as well as three additional regions outside these protected units: the municipalities of Conceição do Araguaia (CA), São Geraldo do Araguaia (SA), and Ourilândia do Norte/São Félix do Xingu (ON/SX). All sites are located in southeastern Pará State, Brazil, within the Eastern Amazon (5°0′–9°0′ S and 48°0′–51°0′ W), as illustrated in Fig 1. The three additional regions were selected based on the presence of *canga* habitats, their proximity to the Carajás Mosaic, and logistical feasibility of fieldwork access.

**Fig 2:**
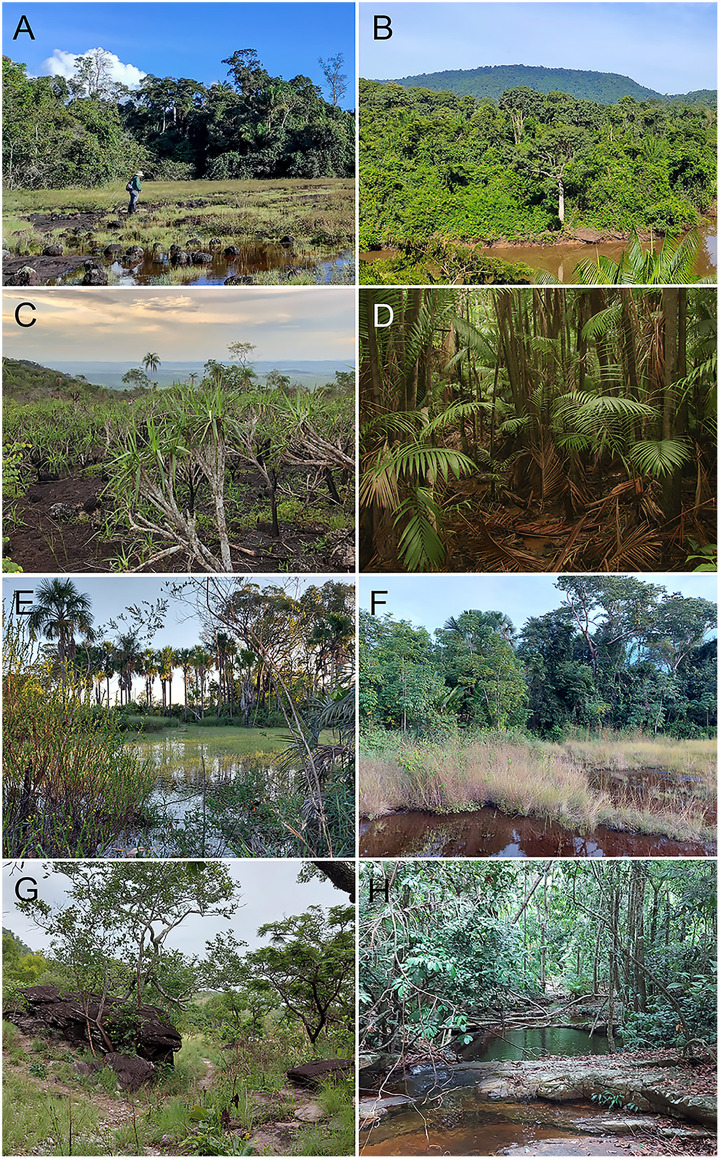
Examples of habitats surveyed in the study area. (A–B) Serra dos Carajás; (C–D) Ourilândia do Norte; (E–F) Conceição do Araguaia; (G–H) São Geraldo do Araguaia. All photographs were taken by the authors: F. M. Silva (A–B); J. F. Sarmento (C); F.J.M. Rojas-Runjaic (D); and A.O. Maciel (E–H). Images are licensed under CC BY 4.0.

The regional climate is classified as Aw (Tropical Savanna) according to the Köppen–Geiger climate classification system [36], with two contrasting seasons: a dry season lasting approximately five months (June–October), with average precipitation around 60 mm in the driest month; and a markedly rainy season (December–April), with precipitation averaging ~ 2,000 mm in the rainiest months. Mean Monthly temperatures range from 23ºC to 26ºC throughout the year [26]. The region’s vegetation includes several prominent typologies, such as dense and open rainforests, alluvial ombrophilous forests, and *Campos Rupestres* (*canga*) that thrive on ironstone outcrops on ridges at altitudes ranging from 280 m to 904 m (average 670 m) [26]. Furthermore, each vegetation type harbors species uniquely adapted to local environmental conditions, including anthropogenic disturbances and seasonal climate variation (Fig 2).

### Sampling methods and species identification

We compiled the species inventory using three data sources. First, we processed records from the herpetological collection of the Museu Paraense Emílio Goeldi (MPEG), in Belém, Pará, Brazil, which included georeferenced data relevant to our study. Second, we incorporated occurrence data from scientific literature. Finally, we included data collected during six field expeditions conducted by the authors between September 2021 and September 2022, covering both rainy and dry seasons across the three areas surrounding the Carajás Mosaic, as previously described. During these expeditions, we surveyed specific sites along *canga* formations, access roads, and anthropized areas, with geographic coordinates recorded for each locality.

We employed field techniques suitable for rapidly assessing extensive areas, following the approach of Crump & Scott [37]. These included Visual Encounter Surveys (VES) and Acoustic Encounter Surveys (AES), conducted during both daytime and nighttime. We inspected various habitats and microhabitats such as ponds, streams, fallen logs, leaf litter, burrows, aquatic vegetation, bushes, trees, and other substrates within *canga* areas and forest–*canga* transition zones. We also used snake hooks and tongs to handle large animals and inspect substrates. Given the predominantly rocky soils in the sampled areas, pitfall traps were not employed.

After sampling, we followed standardized procedures for each specimen, including field identification, recording geographic coordinates, photographic documentation, and euthanasia using size-appropriate protocols. We collected tissue samples (liver or muscle) and preserved them in 2 mL sterile cryogenic tubes with ≥99% ethanol. We fixed the specimens in 10% formalin and later transferred to 70% ethanol for long-term storage. We deposited all voucher specimens and tissue samples in the herpetology collection of the Museu Paraense Emílio Goeldi.

We identified specimens at the species level based on external morphological characters described in original descriptions, redescriptions, and taxonomic revisions. We followed the nomenclature of Frost [38] and Segalla et al. [39] for amphibians, and Guedes et al. [40] for squamate reptiles. We also used bioacoustic characteristics, as described by Köhler et al. [41], for species identification. When species-level identification was uncertain, records were annotated with qualifiers such as “cf.” (to be compared with), “aff.” (similar to), or “sp.” (species unknown), following the guidelines of Sigovini et al. [42]. In cases where morphology alone was insufficient for conclusive identification, we employed DNA barcoding and/or phylogenetic analyses, following Vences et al. [13].

### Call recording and bioacoustic analysis

When we successfully detected and approached calling anuran species, we recorded their vocalizations for bioacoustic diagnosis. If we collected the recorded specimen, it was used as a voucher for the corresponding recordings, following Khöler et al. [41]. If collection was not feasible, we obtained another conspecific specimen from the same location as a reference. We recorded vocalizations using a Samsung J5 Prime smartphone with the RecForge II app (Android), configured to capture calls in WAV format, mono channel, 44.1 kHz sampling rate and 32-bit depth.

Subsequent analyses of call recordings were conducted in Raven Pro 1.3 [43]. We measured note duration, silent intervals between notes, and call duration from oscillograms. Fundamental and dominant frequencies of notes were determined using power spectrum graphs. For spectral analyses, we applied a Blackman window of 20 ms (82 Hz), with an 80% time-grid overlap and a hop size of 176 samples. The frequency grid was set with a DFT size of 2,048 samples. We generated oscillogram and spectrogram illustrations using the *tuneR* 1.4.1 [44] and *seewave* 2.2.0 [45] packages in R 4.2.1 [46].

### Richness and species composition

To compare species composition among study areas, we generated Venn Diagrams using both primary data (collected in this project) and secondary data (museum and scientific collections). We created the diagrams using the *ggvenn* 0.1.9 package in R 4.2.1 [46]. We also calculated similarity among areas using the Jaccard index with UPGMA clustering, implemented in the *vegan* package in R 4.2.1 [46]. The Jaccard index ranges from 0 to 1, with values closer to 1 indicating greater similarity in herpetofauna composition. All analyses were performed for the entire dataset and separately for amphibians and reptiles.

To provide context for potential biases in species richness comparisons among areas, we compiled a summary of sampling effort across data sources in Supplementary Information (Tables 1–2 in S5 File). These tables detail the number of expeditions and sampling days for fieldwork, the number of museum vouchers available, the number of published surveys incorporated, and the genetic sampling effort (individuals and species analyzed) for each study area.

### DNA barcode references

To establish a baseline for genetic diversity of the regional herpetofauna, we used tissue samples from both recent field collections and specimens already stored in the MPEG tissue bank. We extracted Genomic DNA using the DNeasy Blood & Tissue kit (Qiagen, Hilden, Germany), following the manufacturer’s protocol. DNA quality was assessed with 1% agarose gel electrophoresis, and quantification was performed using a Qubit 3.0 fluorometer (Qubit dsDNA HS Assay, Thermo Fisher Scientific) and a NanoDrop™ spectrophotometer (Thermo Fisher Scientific).

We amplified regions of the COI and 16S rRNA genes using two primer sets (S1 Table). Each PCR contained 3 µL of genomic DNA (10–15 ng), 2.5 µL of 10^x^ reaction buffer [100 mM Tris-HCl (pH 8.3, 500 mM KCl], 1.2 µL of 50 mM MgCl_2_, 2 µL of dNTP mix (2 mM each), 0.5 µL of each forward and reverse primer (10 µM; S1 Table), 0.2 U of Taq polymerase (Thermo Fisher), and Milli-Q water to a final volume of 25 µL. Reactions were run in a Veriti 96-Well Thermal Cycler (Thermo Fisher), with cycling conditions specific to each primer set (COI and 16S rRNA; S2 Table). We carried out bidirectional sequencing using the BigDye Terminator v3.1 Cycle Sequencing kit (Thermo Fisher), following a modified protocol [47] on an ABI 3730 DNA Analyzer (Applied Biosystems).

Trace files (*.ab1) were processed in Geneious Prime v2023 (Biomatters Ltd) to generate consensus sequences from forward and reverse reads. To identify potential sequencing errors, we aligned sequences using MAFFT v7.388 [48] with the *Auto* algorithm, performed separately for each marker. When inconsistencies were detected, we re-examined the raw trace files to distinguish true polymorphisms from sequencing errors. To check for possible labeling errors or sample mishandling, we verified the phylogenetic positions of the sequenced specimens using a maximum likelihood (ML) analyses in RAxML v8.2 [49] via the CIPRES portal (http://phylo.org), applying the GTR + G substitution model and rapid bootstrapping with 1,000 replicates (S1 and S2 Figs). Misidentified records were re-examined by checking museum vouchers; problematic sequences were either removed or corrected before continuing analyses.

To evaluate the species identification potential of the DNA barcodes, we applied the Assemble Species by Automatic Partitioning (ASAP [50]) method using four separate alignments (COI and 16S rRNA for amphibians and squamate reptiles, respectively), under the K80 model (ts/tv = 2.0). All sequences generated were submitted to the BOLD Systems (accession numbers ITVHP001–24-ITVHP500–24; https://www.boldsystems.org/) and to GenBank (accession numbers PQ733108-PQ733531).

### Ethical statement

Specimens and tissue samples were collected in accordance with ethical and legal guidelines, under collection permits #79306−3, issued by the Brazilian Federal Agencies Instituto Chico Mendes de Conservação da Biodiversidade (ICMBio) and Instituto Brasileiro do Meio Ambiente e dos Recursos Naturais Renováveis (IBAMA). We did not perform any surgical or experimental procedures on live animals.

## Results

**Fig 3:**
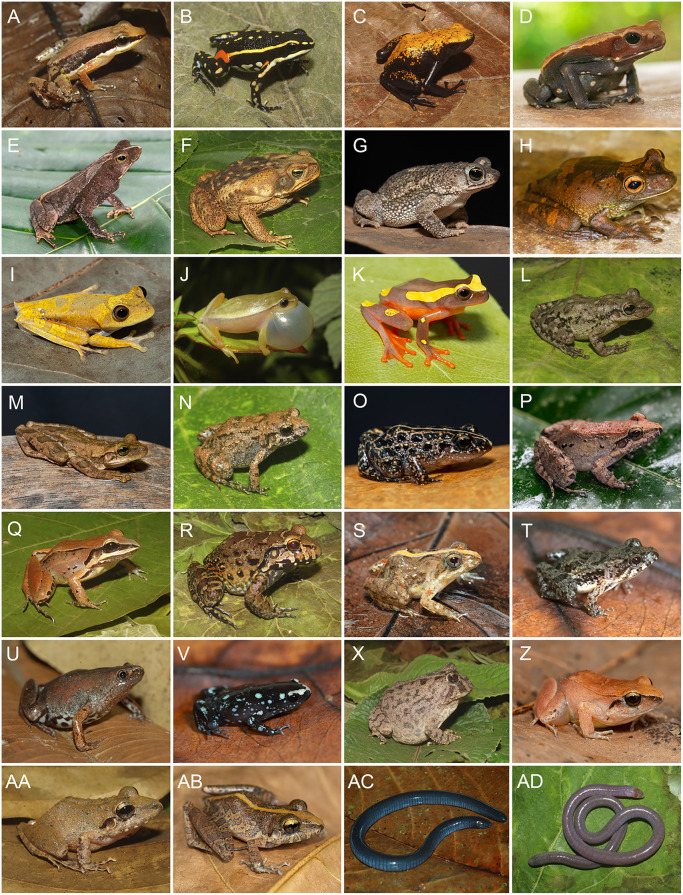
A selection of amphibians recorded in southeastern Pará, Brazil, Eastern Amazon. (A) *Allobates carajas*; (B) *Ameerega flavopicta*; (C) *Adelphobates galactonotus*; (D) *Rhaebo guttatus*; (E) *Rhinella dapsilis*; (F) *Rhinella diptycha*; (G) *Rhinella mirandaribeiroi*; (H) *Boana boans*; (I) *Boana geographica*; (J) *Dendropsophus anataliasiasi*; (K) *Dendropsophus* aff. *triangulum*; (L) *Scinax similis*; (M) *Osteocephalus taurinus*; (N) *Adenomera kayapo*; (O) *Adenomera saci*; (P) *Leptodactylus leptodactyloides*; (Q) *Leptodactylus* aff. *kilombo*; (R) *Leptodactylus vastus*; (S) *Pseudopaludicola canga*; (T) *Pseudopaludicola javae*; (U) *Chiasmocleis avilapiresae*; (V) *Chiasmocleis centralis*; (X) *Proceratophrys* cf. *cristiceps*; (Z) *Barycholos ternetzi*; (AA) *Pristimantis giorgii*; (AB) *Pristimantis moa*; (AC) *Caecilia tentaculata*; (AD) *Microcaecilia* sp. All photographs were taken by the authors: F.J.M. Rojas-Runjaic (A–C, F, J, L, N, Q, R, X, AA, AD); A.O. Maciel (D, G–I, K, M, O, P, R–V, Z, AB, AC). Images are licensed under CC BY 4.0.

**Fig 4:**
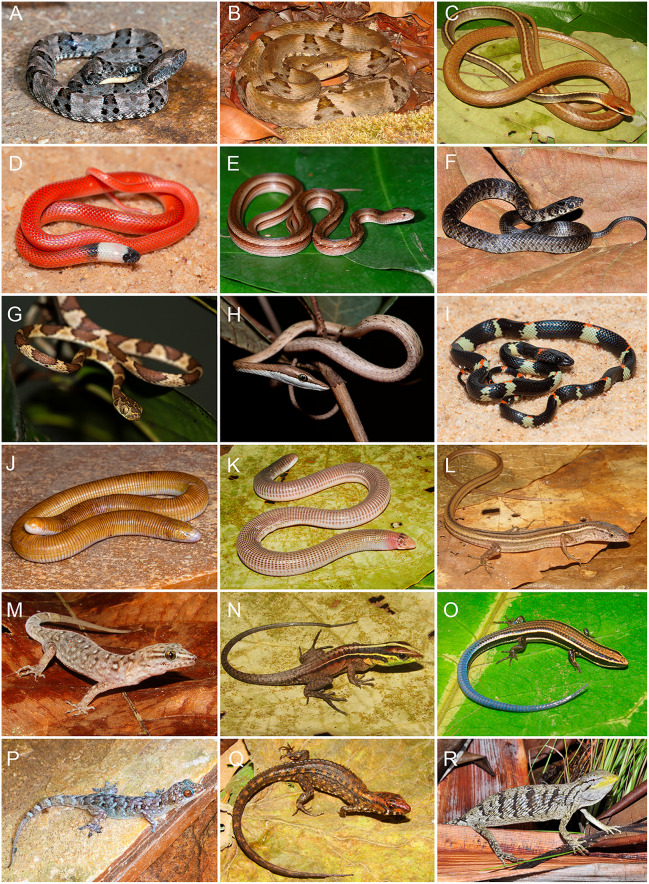
A selection of squamate reptiles recorded in southeastern Pará, Brazil, Eastern Amazon. (A) *Bothrops atrox*; (B) *Bothrops* sp.; (C) *Chironius flavolineatus*; (D) *Drepanoides anomalus*; (E) *Dryophylax hypoconia* 02; (F) *Erythrolamprus carajasensis*; (G) *Imantodes cenchoa*; (H) *Oxybelis aeneus*; (I) *Siphlophis worontzowi*; (J) *Amphisbaena alba*; (K) *Amphisbaena anomala*; (L) *Cercosaura olivacea*; (M) *Gymnodactylus amarali*; (N) *Kentropyx calcarata*; (O) *Micrablepharus atticolus*; (P) *Phyllopezus* aff. *pollicaris*; (Q) *Potamites* aff. *ecpleopus*; (R) *Polychrus acutirostris*. All photographs were taken by the authors: A.O. Maciel (A, D, E, G–J, P); F.J.M. Rojas-Runjaic (B, C, K–O, Q); M.M. dos Santos (F, R). Images are licensed under CC BY 4.0.

We recorded a total of 242 species across the four surveyed areas. Squamate reptiles comprised 59.1% of the assemblage (93 snake species, 43 lizard species, and seven amphisbaenians) while amphibians accounted for the remaining 40.9%, including 93 anurans and six caecilians. Among squamates, Dipsadidae was the most species-rich family, with 50 species (34.9%). Among amphibians, Hylidae was the most representative, with 33 species (33.3%) (Figs 3 and 4). According to the IUCN, 90.6% of the species recorded in southeastern Pará are classified as Least Concern (LC), 1.2% as Data Deficient (DD), and 7.8% have not yet been evaluated (see S3–S4 Tables). A summary of richness per area is provided in Table 1. Detailed species lists, including occurrence records and distribution by area, are available in the Supporting Information (S3–S4 Tables).

**Table 1:** Summary of herpetofauna richness across study areas in southeastern Pará, Brazil, Eastern Amazon.

Area	Total species	Amphibians	Squamate reptiles
Carajás Mosaic (CM)	221	92	129
Ourilândia do Norte/São Félix (ON/SX)	126	54	72
São Geraldo do Araguaia (SA)	96	43	53
Conceição do Araguaia (CA)	63	31	32
**Total**	**242**	**99**	**143**

**Fig 5:**
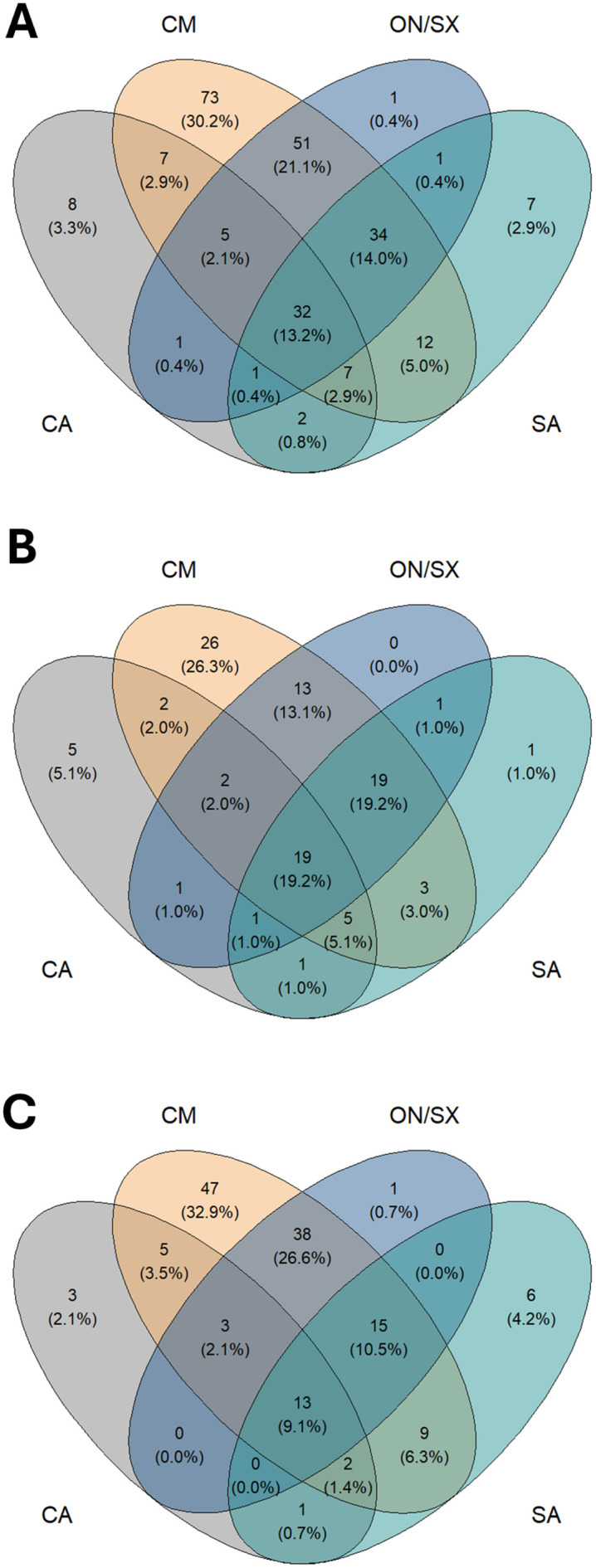
Distribution of exclusive and shared species across sampled areas. (A) All species; (B) amphibians; (C) squamate reptiles. CA: Conceição do Araguaia; CM: Carajás Mosaic; ON/SX: Ourilândia do Norte/São Félix do Xingu; SA: São Geraldo do Araguaia.

The Carajás Mosaic exhibited the highest species richness (221 species), including 73 exclusive to the area and 51 shared with Ourilândia do Norte/São Félix do Xingu. Ourilândia do Norte/São Félix do Xingu followed with 126 species, none of which were exclusive to this area. São Geraldo do Araguaia had 96 species, with seven exclusive and 12 shared with the Carajás Mosaic, whereas Conceição do Araguaia recorded 63 species, including eight exclusive and seven shared with the Carajás Mosaic (Fig 5A). When analyzed separately, amphibian and squamate compositions revealed greater overlap between the Carajás Mosaic and Ourilândia do Norte/São Félix do Xingu (Fig 5B, 5C; S3–S4 Tables).

**Fig 6:**
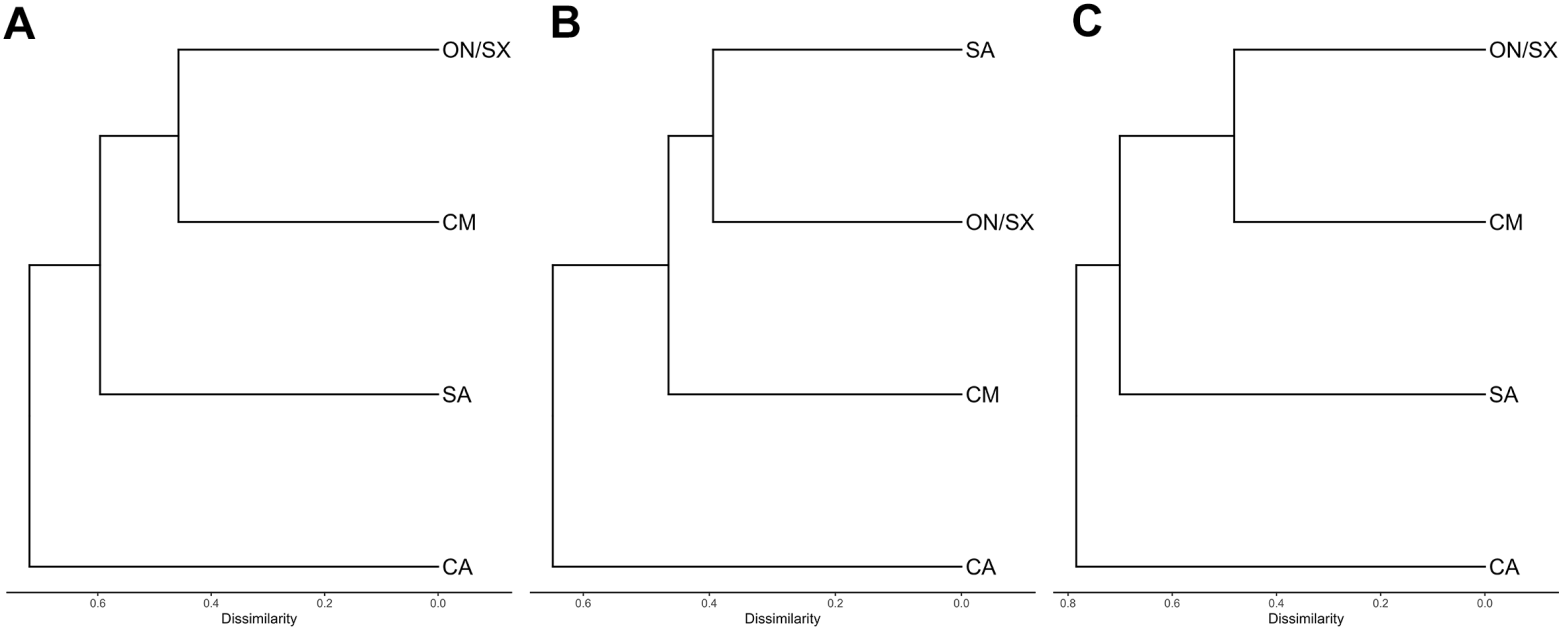
Similarity between the sampled areas. (A) All species, (B) amphibians, and (C) squamate reptiles. CA: Conceição do Araguaia, CM: Carajás Mosaic, ON/SX: Ourilândia do Norte/São Félix do Xingu, SA: São Geraldo do Araguaia.

The Carajás Mosaic and Ourilândia do Norte/São Félix do Xingu showed the highest species similarity (54%), followed by São Geraldo do Araguaia and Ourilândia do Norte/São Félix do Xingu (44%). In contrast, the lowest similarity was observed between Conceição do Araguaia and the Carajás Mosaic (21.8%) (Fig 6A; Table 1 in S1 File). When analyzed separately, the amphibian assemblage showed the highest similarity between Ourilândia do Norte/São Félix do Xingu and São Geraldo do Araguaia (60.6%), followed by the Carajás Mosaic and Ourilândia do Norte/São Félix do Xingu (57.8%), whereas Conceição do Araguaia and the Carajás Mosaic again showed the lowest similarity (28.8%) (Fig 6B; Table 2 in S1 File). Squamate reptiles exhibited the same clustering pattern as the combined dataset of amphibians and reptiles (Fig 6C; Table 3 in S1 File).

### New records, range extensions, and taxonomic remarks

Although previous studies had documented most species observed during our field expeditions, we confirmed ten species as new records for the state of Pará (nine amphibians and one snake) and identified two range extensions. We based these confirmations on historical specimens and previously published records (S3 and S4 Tables). During fieldwork, we recorded advertisement calls from several anuran species, which were crucial for confirming identifications, particularly for *Adenomera saci*, *Allobates carajas*, and *Dendropsophus anataliasiasi*. Below, we provide a summary of each new record, including details on species identification, biology, distribution, and materials examined. The summaries are organized alphabetically, starting with amphibians, followed by squamate reptiles.

### Amphibians

#### Adenomera saci.

This terrestrial and nocturnal frog has a broad distribution across central-western and northeastern Brazil, ranging from northern Mato Grosso do Sul through eastern Mato Grosso, Goiás, and Tocantins to southern Maranhão. Its habitat includes the Cerrado and transitional environments leading to the Amazon basin [51–53]. Although its external morphology is nearly indistinguishable from *A. martinezi* (Fig 3), they can be differentiated by bioacoustic features. *Adenomera martinezi* produces pulsed notes, while *A. saci* produces tonal notes [51]. See Fig 1 in S2 File for details. The call parameters observed are consistent with those previously described by Carvalho and Giaretta [51], providing further confirmation of the species identity of our collected specimen from Conceição do Araguaia. Notably, this finding represents the first record of *A. saci* in Pará, expanding its known distribution by approximately 104 km westward from Colinas do Tocantins, Tocantins, the nearest previously recorded locality [51,52].

#### Allobates carajas.

This is a diurnal, ground-dwelling frog species endemic to Pará, known only from a few locations within well-preserved Amazonian rainforests in the Serra dos Carajás [25,54]. Molecular evidence has revealed that *A. carajas* is sister to *A. crombiei*, its geographically closest and morphologically most similar congener [54,55]. Despite their pronounced phenotypic similarity, these two species exhibit significant genetic divergence, with an uncorrected p-distance of 5% based on the 16S rRNA DNA barcode [54]. Additionally, they can be readily diagnosed by finger IV morphology and acoustically by the number of notes per call and inter-note duration within calls [54,56]. We recorded the call of a single male in São Felix do Xingu which has temporal and spectral call parameters reported consistent with those described by Simões et al. [54] (see Fig 2 in S2 File). Additionally, we sequenced some specimens collected from São Geraldo do Araguaia (e.g., MPEG 43889 and 43891). The 16S sequences differed by 0.5% to 1.1% from a paratopotype of *A. carajas* from Parauapebas, Pará (GenBank accession code: MK060055.1), analyzed by Simões et al. [54]. These data evidence the first confirmed locality records for *A. carajas* outside the Carajás National Forest. We highlight that the record of the species from UHE Belo Monte, Vitória do Xingu, north of the Xingu River, reported by Réjaud et al. [55] is unreliable. The available 16S rRNA sequence for that specimen (GenBank accession code MT627183) diverges by 2.0–2.2% from all other known homologous sequences of *A. carajas*, including the type series.

#### *Atelopus* sp.

Specimens of *Atelopus* have been collected in the Carajás region over the past decades and have been assigned to two names: *A. spumarius* (e.g., [32]) and *A. hoogmoedi* (e.g., [25,57]). Currently, *A. spumarius* is restricted to Peru [58], while species of the *A. hoogmoedi* complex are distributed along the Guiana Shield, and in areas within the Tapajós center of endemism [58,59]. However, to the best of our knowledge, only a single individual from the Xingu center of endemism, which encompasses our study area, has been sequenced and included in a phylogeny of the genus [60]. This individual, collected in the municipality of Anapu, state of Pará, is located more than 200 km from the Carajás region and has been identified as a candidate species or *Atelopus* sp. [60,61]. Given that the species from our study area has not yet been included in phylogenetic analyses, we have opted to refer to it as *Atelopus* sp., pending future phylogenetic assessment.

#### Barycholos ternetzi.

This small species of Strabomantidae (Fig 3) is primarily found in forested environments, though it can also occur in open vegetation formations [53], across the states of São Paulo, Minas Gerais, Goiás, Mato Grosso, Tocantins, and Maranhão [62–64]. A karyotypic study suggested potential genetic differentiation between southern and northern populations of *B. ternetzi* [64]. More recently, a molecular study [65] published in a conference abstract provided phylogenetic evidence supporting two distinct lineages with significant genetic divergence: one grouping specimens from the north and northwestern, and another from the south and southeastern of the species’ distribution. The 16S sequences of two of our collected individuals (MPEG 44016 and 44017) differ by 0.27% and 0.6%, respectively, from the sequence of the *B. ternetzi* individual (CFBHT11284) (GenBank accession code: MT117876.1), which originates from the locality closest to the type locality available in GenBank. In contrast, they differ by 1% and 1.5% from another *B. ternetzi* individual (CFBH-T 306) (GenBank accession code: DQ283094.1) from Gurinhatã, Minas Gerais, which represents the southernmost sampled locality in GenBank. Our record, located closer to the type locality (“Maranhão River, Goiás, Brazil”) than the southern populations, is significant as it represents the first occurrence of *B. ternetzi* in Pará.

#### Chiasmocleis centralis.

Previously considered endemic to the Cerrado [66], this small fossorial frog was thought to be restricted to its type locality in Goiás [53,67]. However, De Sá et al. [66] included specimens from Maranhão and Tocantins in their phylogenetic analyses, and Arantes [68] expanded its known range to 25 localities across Goiás, Tocantins, Mato Grosso, Minas Gerais, and Distrito Federal, corroborated by molecular data. *C. centralis* is part of the *C. albopunctata* species group, which is considered a species complex due to cryptic morphology and unclear genetic diversity [66,68]. We collected a juvenile of *C. centralis* (MPEG 44026, SVL: 16.3 mm) in Conceição do Araguaia (Fig 3), southeastern Pará (−8.229444°, −49.783861°; 220 m asl). Genetic analysis of the 16S rRNA fragment showed low uncorrected pairwise genetic distances (0.2–0.6%) between our specimen and others from Tocantins and Goiás [66,68] (0.2% with MPEG 29414 from Araguatins, Tocantins and 0.6% with CHUNB 51830 from Carolina, Goiás), confirming conspecificity with *C. centralis*. This is the first record of *C. centralis* in Pará, extending its known distribution approximately 120 km from Caseara and 185 km from Curicaca, Tocantins [68].

#### Dendropsophus anataliasiasi.

This small nocturnal tree frog is commonly found in open Cerrado environments (Fig 3), perching on low vegetation near permanent lentic water bodies [53]. It is distributed across central Brazil, including Maranhão, Tocantins, Goiás, and Mato Grosso [53,69,70]. Phylogenetically, *Dendropsophus anataliasiasi* is sister to *D*. *elianeae* and is part of a clade that includes *D. sanborni* and *D. rubicundulus* [70]. The species can be easily distinguished from these congeners, as well as from *D. cachimbo* (a geographically and phylogenetically close congener) by its bioacoustics [71–74]. Additionally, *D. anataliasiasi* lacks a supratympanic fold, has a subacuminate snout in dorsal view, and a head longer than wide, which differentiate it from its close relatives [69]. Vocalizations recorded in this study agreed in most spectral and temporal attributes with those described by Teixeira and Giaretta [71] for topotypes of *D. anataliasiasi* from Tocantins (see Fig 3 in S2 File). Morphologically, our specimens also matched *D. anataliasiasi* [69] in having a head longer than wide (HL/HW = 1.1–1.2), head width equivalent to 3.9–4.1 times SVL, and a similar overall color pattern. This represents the first record of *D. anataliasiasi* for Pará, extending its known distribution approximately 163 km west of Nova Olinda, Tocantins, the nearest previously recorded locality [71].

#### *Dendropsophus* aff. *minutus.*

Gehara et al. [75] demonstrated that *Dendropsophus minutus*, once considered widely distributed across South America, is actually a species complex comprising at least 25 deep mitochondrial lineages, some of which may represent undescribed species. *Dendropsophus minutus* sensu stricto appears to be restricted to its type locality in the Atlantic Forest of southeastern Brazil. Although Cassundé et al. [25] included *D. minutus* in the amphibian fauna of Pará, Gehara et al. [75] suggested, based on genetic divergence, that populations from southeastern Pará may correspond to an undescribed species. Further studies incorporating genetic, bioacoustic, and morphological data are necessary to clarify the taxonomic status of the southeastern Pará lineages. Until then, we refer to these populations as *Dendropsophus* aff. *minutus*.

#### *Dendropsophus* aff. *nanus.*

Cassundé et al. [25] and Pinheiro et al. [32] included *Dendropsophus nanus* in their lists of amphibians of Pará and southeastern Pará, respectively. A recent study by Seger et al. [76] has provided new insights into the taxonomic *status* of *D. nanus*. Seger et al. [76] conducted a comprehensive analysis incorporating molecular, morphological, and bioacoustic data, along with a broad geographic sampling of the *D. nanus-D. walfordi* complex. Their findings demonstrated that *D. nanus sensu stricto* is restricted to northern Argentina, Paraguay, south and central-western Brazil, where it is sister to a clade comprising *D. walfordi* and a candidate species referred to as Lineage C. Moreover, Seger et al. [76] revealed that populations previously identified as *D. nanus*, distributed across southeastern and northeastern Brazil, including southeastern Pará, actually belong to a morphologically and acoustically cryptic lineage. This lineage is phylogenetically close to, but not sister to, *D. nanus sensu stricto*, which was referred to by Seger et al. [76] as Lineage B. We refer to this lineage as *D.* aff. *nanus*, highlighting the urgent need for further studies to test the hypothesis proposed by Seger et al. [76] and to confirm whether the populations in southeastern Pará correspond to this undescribed cryptic species.

#### *Dendropsophus* aff. *triangulum.*

While Pinheiro et al. [32] listed *D. leucophyllatus* from several localities in southeastern Pará, recent molecular evidence has provided valuable insights into the taxonomic status of this species [77,78]. These studies indicate that *D. leucophyllatus sensu stricto* is limited to the Guiana Shield, north of the Amazon River. Despite some topological incongruences between the phylogenetic hypotheses of Caminer et al. [77] and Pirani et al. [78], both agree that specimens of the *D. leucophyllatus* group from southeastern Pará represent at least two independent evolutionary lineages more closely related to *D. triangulum* than to *D. leucophyllatus* (i.e., clades D and G in [77]; and “*D. leucophyllatus* Central” in [78]). Given this evidence, we designate the specimens from southeastern Pará attributable to this species group as *Dendropsophus* aff. *triangulum*. This nomenclature highlights that they likely correspond to a candidate species distinct from both *D. leucophyllatus* and *D. triangulum.*

#### Hyalinobatrachium iaspidiense.

**Fig 7:**
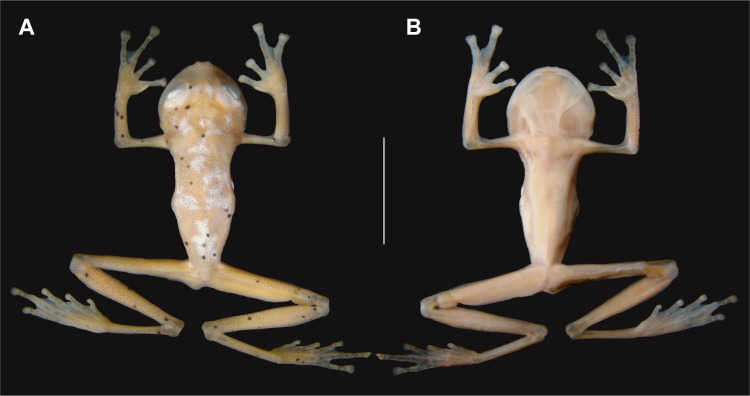
*Hyalinobatrachium iaspidiense* (MPEG 42347). (A) Dorsal and (B) ventral views of an adult male (SVL: 20.0 mm) from Serra Norte, Carajás National Forest, Pará state. Scale bar = 10 mm. Photos by FJMRR (CC BY 4.0).

This small glassfrog is widely distributed across lowland and upland tropical rainforests of the eastern Guiana Shield and Amazon in Ecuador, Peru, Venezuela, Guyana, Suriname, French Guiana, and Brazil [79]. In Brazil, it has been reported only from a few localities in Amapá [80,81], northern Pará [22], northeastern Amazonas [82], and northern Mato Grosso [83]. *Hyalinobatrachium iaspidiense* inhabits forested streams, where it is typically detected at night vocalizing from the underside of leaves in the lowest forest strata [84]. This species can be readily distinguished, even in preservative, from all other species of *Hyalinobatrachium* (except for *H. tricolor* and *H. mesai*) by having large irregular iridophore spots on the dorsum. *Hyalinobatrachium tricolor* and *H. mesai* have similar spots and are indistinguishable from *H. iaspidiense* based on external morphology, but both are restricted to the Guiana region north of the Amazon River [79,85]. During our review of specimens deposited in the herpetology collection of the MPEG, we identified two previously unrecognized glassfrogs corresponding to *H. iaspidiense* (MPEG 42347 and MPEG 43023) (Fig 7). Both were collected in Serra Norte, Carajás National Forest, at the following coordinates: MPEG 42347 (−6.076071°, −50.200262°, 475 m asl.) on March 1, 2019; and MPEG 43023 (−6.086480°, −50.113020°, 434 m asl.) on February 19, 2020. This discovery represents the second record for Pará [22] and the easternmost records for the species [80]. The new locality extends the distribution ca. 742 km northeast of the lower Cristalino River (Mato Grosso), about 784 km southeast of the Reserva Extrativista Beija-Flor Brilho de Fogo (Amapá), and approximately 962 km southeast of ESEC Grão-Pará (Pará), which are the nearest and previously easternmost known localities for the species [22,81,83].

#### Leptodactylus podicipinus.

This species belongs to the *Leptodactylus melanonotus* group, which has posed significant challenges for South American amphibian systematists over the past decades due to the inclusion of several widely distributed species with similar morphology [86,87]. *L. podicipinus* is broadly distributed in open formations across Argentina, Bolivia, Brazil, and Paraguay. In Brazil, its range extends through the dry diagonal from the central Cerrado to southern formations. Within the Amazon, it has been recorded in areas adjacent to the Madeira and Guaporé Rivers [87,88]. The closest known records to our study areas are in northern Tocantins, within the Tocantins-Araguaia interfluve. We sequenced four specimens collected in Conceição do Araguaia (MPEG 44060–44062, 44064). The 16S sequences differed by 1.6% –1.9% from an individual of *L. podicipinus* from Cuiabá, Mato Grosso (GenBank accession code: MW291351.1), analyzed by Gazoni et al. [87], and by 0% –0.25% from an individual from Estreito, Maranhão (GenBank accession code: KU495350.1), analyzed by Lyra et al. [89]. Thus, our study provides the first record of the species in the state of Pará.

#### *Leptodactylus* aff. *kilombo.*

The *Leptodactylus mystaceus* complex was recently revised by Silva et al. [90]. Based on the geographical distribution of the lineages identified in that study, we observed the potential occurrence of two lineages in our study areas: the first is *Leptodactylus mystaceus sensu stricto*, and the second is identified as *Leptodactylus* cf. *mystaceus*. We sequenced three specimens collected from São Geraldo do Araguaia (MPEG 36362, 43901, 43902) and two from São Félix do Xingu (MPEG 39549, 41760). These sequences showed a closer genetic affinity to individuals of the *L*. cf. *mystaceus* lineage described by Silva et al. [90]. For instance, our sequences exhibited a genetic divergence of 0.48% –0.72% compared with an individual (CFBHT12181) of *L*. cf. *mystaceus* from Marabá, Pará (GenBank accession code: MT117876.1), as analyzed by Silva et al. [90]. Consequently, based on these five specimens, we confirm the presence of the *L.* cf. *mystaceus* lineage in addition to *L. mystaceus* in our species list. However, we updated the nomenclature to *L*. aff. *kilombo*, as this represents a sister lineage to *L. kilombo* and is more closely related to that species than to *L. mystaceus* (see Fig 2 in [90]).

#### Pithecopus araguaius.

This species of leaf frog was originally described from areas within the Cerrado biome in the state of Mato Grosso, Brazil, and was phylogenetically positioned as the sister species to *Pithecopus hypochondrialis* [91]. In the state of Pará, it was mentioned in a review of the *P. hypochondrialis* complex, which identified individuals from a locality east of the Tocantins River, in the municipality of Marabá, as belonging to the *P. araguaius* clade, along with individuals from the type locality in Mato Grosso and two localities in Colombia [92]. During our expeditions, we encountered eight individuals in Conceição do Araguaia (MPEG 43621 and 44075), São Félix do Xingu (MPEG 43973–43976, 43978), and São Geraldo do Araguaia (MPEG 43913), three localities within the range defined for the species in Fig 2 of Magalhães et al. [92]. The 16S sequences showed a divergence of 3.46% –4.2% compared with a paratopotype of *P. araguaius* (ZUEC 21659) from Pontal do Araguaia, Mato Grosso (GenBank accession code: MF926298.1). Furthermore, the sequences differed by 0.25% –1.23% from those of an individual (MZUSP 140079) from Marabá, Pará (GenBank accession code: OQ916232.1), analyzed by Magalhães et al. [92]. Thus, our data confirm the presence of *P. araguaius* in southeastern Pará.

#### Physalaemus centralis.

This species belongs to the *Physalaemus cuvieri* group and is widely distributed across the Brazilian Cerrado [93]. We sequenced one specimen from Conceição do Araguaia (MPEG 44072). The 16S sequence of this specimen differed by 0.75% from that of an individual of *P. centralis* from Wanderlândia, Tocantins (GenBank accession code: MW201201.1), and by 0.75% from that of an individual from Chapada dos Guimarães, Mato Grosso (GenBank accession code: MW201199.1). Both sequences were analyzed by Loebmann et al. [94] in a niche modeling study of the species. Thus, our record represents the first documented occurrence in the state of Pará.

#### Pristimantis moa.

This small nocturnal rainfrog inhabits forested environments in the Cerrado biome, where it can be found both on the ground and perched on low vegetation [95]. The known geographic distribution of this species seems to be very restricted and, until now, it was known only from a few localities in northern Tocantins and southwestern Maranhão [95,96]. Phylogenetic evidence indicates that *Pristimantis moa* is the sister species of *P. giorgii*, its geographically close congener and the most common *Pristimantis* in southeastern Pará. Both species are almost indistinguishable in morphology and advertisement calls, but can be diagnosed using molecular evidence [95,96]. We collected a large series of *Pristimantis* belonging to the *P. conspicillatus* species group from several localities along the Serra das Andorinhas (São Geraldo do Araguaia, Pará). We determined the species identity from a 16S gene fragment of specimen MPEG 42392, collected in Serra das Andorinhas (−6.218917, −48,435972; 125 m a.s.l.) on November 11, 2021. The uncorrected pairwise distance for the 16S gene between specimen MPEG 42392 and *P. moa* from Araguaína in Tocantins and Carolina in Maranhão (GenBank accession codes: KX242518 and KT221610, respectively) was only 0.3%, indicating conspecificity. Additionally, our specimen of *P. moa* (MPEG 42392) from Serra das Andorinhas diverged by 3.0–3.2% from specimens of *P. giorgii* from Tucuruí and Marabá, Pará (GenBank accession codes: KX242516 and KX242515, respectively). These p-distance values are higher than those reported by Oliveira et al. [95] between *P. moa* and *P. giorgii*, corroborating that MPEG 42392 is not conspecific with *P. giorgii*. This represents the first record of *Pristimantis moa* for the State of Pará and the northwesternmost locality for the species, extending its known distribution approximately 108 km NNW of Araguaína (Tocantins), the nearest previously recorded locality [95,96].

#### Pseudopaludicola javae.

This diminutive leptodactylid frog was recently discovered and described from the Araguaia –Tocantins interfluve in western Tocantins, Brazil, an ecotonal zone between the Amazon and Cerrado biomes [97]. It is a vespertine and nocturnal ground-dwelling frog typically found on the soaked soil of seasonally flooded open areas dominated by grasses. However, it also thrives in anthropized environments such as cattle pastures, road margins, and crop fields [97]. We found a total of 12 individuals during our field trips to Conceição do Araguaia (−8.229040°, −49.784710°; 217 m a.s.l.) in March 2022. Several specimens were located based on their advertisement calls and were found on soaked soil along the ecotone between a forest patch and a seasonally flooded open area. On March 17, 2022, we recorded the calls of a single male (MPEG 44093) at 16:34 h. The calls of this specimen (n = 3) consisted of a series of 11–18 pulsed notes, emitted at a rate of 51.3–55.0 notes/min. Note duration ranged from 297 to 339 ms (averaging 321 ± 12 ms; n = 18), with 10–12 pulses per note emitted at a rate of 32.2–36.5 pulses/sec. Dominant frequency corresponds to the fundamental and ranged from 4.91 to 5.21 kHz (average 5.04 ± 0.1 kHz; n = 18). All the temporal and spectral call parameters documented for MPEG 44093 are consistent with those described by Silva et al. [97]. In addition to the acoustic evidence, we confirmed the species identity of our specimens from Conceição do Araguaia with molecular data. The uncorrected pairwise distance for the 16S gene between specimen MPEG 44094 and the type series of *P. javae* (GenBank accession codes: OR509824–OR509828) was only 1.3%. This represents the first record of *Pseudopaludicola javae* in Pará and the northernmost locality for the species, extending its known distribution approximately 183 km north of Fazenda Escondida (type locality) in Tocantins [97].

#### Pseudopaludicola mystacalis.

This small frog, found in lentic water bodies across open formations of South America, belongs to a species complex with at least ten recognized lineages, which diverge genetically by up to 5% [98]. In Pará, the species had previously been recorded only in the municipality of Primavera, in the northeast of the state [98]. We sequenced three specimens from São Geraldo do Araguaia, previously identified as *P. canga* (MPEG 43920–43922). All the 16S sequences of these individuals clustered with *P. mystacalis* sequences. The 16S sequences differed by 0.27% from *P. mystacalis* specimen from Babaçulândia, Tocantins (GenBank accession code: KU495494.1), and by 4% from an individual from Cuiabá, Mato Grosso (GenBank accession code: KF146983.1). Consequently, based on these three specimens, we confirm the presence of *P. mystacalis* in our species list. *Pseudopaludicola mystacalis* and *P. canga* are externally very similar species, with the most notable differences found in their osteological characters [99]. In fact, *P. canga* was originally described based on specimens previously identified as *P. mystacalis* [100].

#### The *Leptodactylus pentadactylus* group.

The *Leptodactylus pentadactylus* group consists of medium- to large-sized frog species distributed mostly across South America [101]. In southeastern Pará, we recorded four species from this group: *Leptodactylus labyrinthicus*, *L. paraensis*, *L. pentadactylus*, and *L. rhodomystax*. *Leptodactylus labyrinthicus* and *L. paraensis* are considered very similar, lacking consistent morphological characteristics to reliably differentiate them [101], which leads to considerable confusion in field identification. We sequenced one specimen from São Geraldo do Araguaia previously identified as *L. labyrinthicus* (MPEG 43900), one specimen identified as *Leptodactylus* sp. (MPEG 43962), and nine specimens identified as *L. paraensis* from São Félix do Xingu (MPEG 43963–43969, 44144, 44145), along with one individual identified as *L*. cf. *paraensis* from Conceição do Araguaia (MPEG 44055). All 16S sequences from these individuals clustered with *Leptodactylus vastus* sequences. The 16S sequences differed by 0.73% –2.18% from an individual of *L. vastus* from Guarabira, Paraíba, Brazil (GenBank accession code: KU495363.1), and by 0.24%– 1.7% from an individual from Los Lagos, Bolivia (GenBank accession code: KF723204.1). Consequently, based on these specimens, we confirm the presence of *L. vastus* in our species list. *Leptodactylus vastus*, also part of the *L. pentadactylus* group, has a disjunct distribution, occurring widely in open formations across northeastern Brazil and with a population in the Bolivian Amazon forest [38]. Although our genetic sample is limited, we consider the possibility that *L. paraensis* may be synonymous with *L. vastus* and that *L. labyrinthicus* might not occur in the state. However, given the complex taxonomy of this group, we expect that broader and more integrative studies will clarify these issues in the future. Therefore, we have retained the previously known species in our list, while adding *Leptodactylus vastus* as the first documented record for Pará.

### Squamata

#### *Dryophylax hypoconia* 02.

As reported by Trevine et al. [102], the diversity within *Dryophylax hypoconia* is underrepresented, consisting of four distinct snake lineages. The first lineage is found in Argentina and southern Brazil (Rio Grande do Sul); the second includes specimens from northern central Brazil (Tocantins); the third is from southeastern Brazil (Minas Gerais and São Paulo); and the fourth includes individuals from plateau regions in Santa Catarina and Rio Grande do Sul. The second lineage occurs on both sides of the Tocantins River and is the sister group to a clade containing species from the Guiana Shield (*D. chimanta*) and the Caribbean (*D. gambotensis* and *D. paraguanae*). We designated the *D. hypoconia* specimens collected in Canaã dos Carajás, Conceição do Araguaia, and São Geraldo do Araguaia, Pará, during our expeditions as *D. hypoconia* 02, following Trevine et al. [102]. We maintain this nomenclature, highlighting that it may represent an undescribed taxon. These specimens represent the first record of *D. hypoconia* in Pará, extending the species’ known distribution approximately 285 km northward.

#### *Phyllopezus* aff. *pollicaris.*

*Phyllopezus pollicaris* is a complex of gecko species widely distributed across the dry diagonal of South America [103]. In a phylogenetic study, Werneck et al. [104] recovered a high genetic diversity within this taxon, revealing several geographically structured cryptic lineages associated with three biomes: Chaco, Cerrado, and Caatinga. The eight well-supported clades identified by these authors may represent distinct species. Among the Cerrado lineages, the one referred to as clade II includes specimens from Serra dos Martírios and São Geraldo do Araguaia, Pará. The specimens we collected in Serra das Andorinhas (São Geraldo do Araguaia) were designated as *Phyllopezus* aff. *pollicaris*, as they likely represent lineage II recovered by Werneck et al. [104]. Following these results, additional analyses of *Phyllopezus* have been conducted, although the taxonomy of the candidate species has not yet been resolved [103,105].

### DNA barcoding references

Our database includes 860 DNA barcodes, comprising 436 COI and 424 16S rRNA sequences obtained from 500 specimens (384 amphibians, 85 lizards, 30 snakes, and one amphisbaenian) previously identified. Most COI sequences ranged from 450–510 bp, while 16S rRNA sequences ranged from 400–430 bp. Overall, the specimens represent 93 well-recognized species, including 50 amphibians, 20 lizards, 22 snakes, and one amphisbaena (Tables 1 and 2 in S3 File). Approximately 40.1% of these species were supported by at least one reference barcode.

Our COI dataset covered a broad taxonomic range, encompassing 27 families (11 amphibians, eight lizards, seven snakes, and one amphisbaenian), 59 genera (22 amphibians, 18 lizards, 18 snakes, and one amphisbaenian) and 87 well-recognized species (48 amphibians, 19 lizards, 19 snakes, and one amphisbaenian). For 16S rRNA, coverage was similar, including 25 families (nine amphibians, eight lizards, seven snakes, and one amphisbaenian), 57 genera (20 amphibians, 19 lizards, 17 snakes, and one amphisbaenian), and 88 well-recognized species (49 amphibians, 20 lizards, 18 snakes, and one amphisbaenian). Compared with GenBank (as of September 14, 2024), our data contributed five new COI records (8.8% of total COI barcodes generated) and two new 16S rRNA records (3.5%) for amphibians. For squamates, contributions were higher, with 25 new COI records (59.5%) and 13 new 16S rRNA records (31.7%) (S5 Table).

In addition to well-recognized species, we sequenced several unconfirmed or candidate species, denoted with ‘aff.’, ‘cf.’, ‘gr.’, or ‘sp.’ Approximately 18% of amphibian barcodes fall into this category, including *Dendropsophus* aff. *minutus, D.* aff. *nanus, D.* aff. *triangulum, Leptodactylus* aff. *kilombo, Microcaecilia* sp., *Physalaemus* aff. *cuvieri, Proceratophrys* cf. *cristiceps, Scinax* aff. *cruentomma,* and *Scinax* gr. *ruber*. For squamates, two unconfirmed species were identified among lizards (*Phyllopezus* aff. *pollicaris* and *Potamites* aff. *ecpleopus*) and one among snakes (*Bothrops* sp.), representing 7.0% of reptile barcodes.

ASAP analyses using both markers revealed largely similar group subdivisions, although the number of distinct lineages differed between genes (S3–S6 Figs; Tables 1–4 in S4 File). Overall, COI recovered more groups than 16S rRNA, with the best ASAP scores yielding 60 and 53 groups among amphibians for COI and 16S rRNA, respectively (Tables 1 and 2 in S4 File), and 44 and 43 groups among squamates (Tables 3 and 4 in S4 File). Threshold distances also differed: for squamates, COI groups were delimited at 8.61% versus 2.53% for 16S rRNA, whereas for amphibians 16S rRNA had a higher threshold (7.73%) compared with COI (5.80%).

To evaluate marker performance, we used the ASAP score, where lower values indicate better species delimitation. For amphibians, 16S rRNA yielded a slightly better score (4.0 vs 5.0), suggesting stronger partitioning. In contrast, for reptiles, COI performed significantly better (3.5 vs. 7.5), indicating more robust lineage delimitation (Tables 1–4 in S4 File).

Marker comparisons revealed differences in phylogenetic resolution for specific taxa. For *Pristimantis*, both markers recovered meaningful groupings, but COI detected finer structure. For example, 16S rRNA grouped *P. giorgii* and *P. moa* into a single lineage (group 5), with *P. latro* in an independent lineage (group 6) (Table 1 in S3 File; S3 Fig). In contrast, COI resolved three subgroups: *P. giorgii* split into two lineages (groups 5 and 6), while *P. moa* (group 44) and *P. latro* (group 24) were separated as species (Table 1 in S3 File; S4 Fig). A similar pattern was observed in *Rhinella diptycha*: 16S rRNA grouped all records into one cluster (47), whereas COI separated them into two (47 and 48) (Table 2 in S3 File; S5 and S6 Figs). For taxa with uncertain classification, such as *Potamites* aff. *ecpleopus,* both markers suggested the presence of two distinct lineages (COI: groups 37 and 38; 16S rRNA: groups 38 and 42) (Table 2 in S3 File; S5 and S6 Figs).

## Discussion

Our results indicate that southeastern Pará harbors high diversity of amphibians and squamate reptiles, with Hylidae and Dipsadidae being the most diverse families. This finding is consistent with previous studies in the Brazilian Amazon, which identified these families as the richest in anuran and snake species [5,22,39,106]. Notably, we recorded a remarkable diversity of snakes, representing 21% of all snake species documented in Brazil [39]. Several authors have emphasized the high snake diversity across the Amazon, reinforcing the importance of biological inventories to clarify distribution patterns for this secretive group [e.g., 5,6,107,108].

The high species richness observed in the Carajás Mosaic and in Ourilândia do Norte/São Félix do Xingu likely reflects the well-preserved state of these areas. The strong similarity in species composition between (75 shared species) can be attributed to their geographic proximity and ecological similarity [109,110]. Protected areas, including conservation units and indigenous lands, play a critical role in safeguarding biodiversity [108,111]. Maintaining large forest remnants remains essential to sustaining viable populations and ecological processes.

Ourilândia do Norte and São Félix do Xingu, located within the Xingu endemism area, have experienced considerable environmental impacts. However, their proximity to the Xikrin Indigenous Land along the Cateté River ensures landscape connectivity with this protected territory. Castro et al. [112] demonstrated that this Indigenous Land, along with other large protected areas, exhibits high connectivity potential. Such connectivity is vital, as forest remnants function as refuges and ecological corridors for species dispersal [113]. In fragmented landscapes, small habitat patches can also act as stepping stones, maintaining connectivity with larger fragments. Recent studies highlight their importance for sustaining functional connectivity in Amazonian lizard populations [114,115]. This underscores the value of protected areas for conserving forest-dwelling species, whereas species adapted to *canga* naturally occur in fragmented and spatially isolated patches.

Our study also expands knowledge of species richness in Pará, raising the number of documented amphibians from 195 to 204 [25] and squamate reptiles from 278 to 279 [39]. The ten new records we report underscore how incompletely surveyed the Amazonian herpetofauna remains. These findings emphasize the need for continued inventories refine taxonomic knowledge and species distribution [106,116–118]. Several new amphibian records were confirmed by vocal analyses (e.g., *Adenomera saci*, *Allobates carajas*, *Dendropsophus anataliasiasi*), demonstrating the value of bioacoustics as a reliable, non-invasive tool for species identification [119]. Acoustic approaches enhance monitoring capacity across habitats and provide insights into ecology, reproductive behavior, and geographic distribution.

The establishment of DNA barcode libraries is also essential for species identification and resolving taxonomic uncertainties, particularly in cryptic taxa. In our study, 58% of amphibian specimens could only be reliably identified through DNA barcoding. Notably, many belonged to taxa with unresolved taxonomy or species complexes, such as *Rhinella* (margaritifera and marina groups) and *Scinax*. For example, the *R. marina* group includes 11 taxa, and recent studies suggest mitochondrial introgression and hybridization with *R. diptycha* south of the Amazon River [120]. Our analysis confirmed that specimens initially identified as *R. marina* were in fact *R. diptycha*.

The taxonomy of *Scinax* has long been problematic due to morphological similarity among species [121]. The name *S. ruber* has been widely applied to diverse Amazonian populations, though molecular studies identified multiple lineages within this group [122]. In our dataset, specimens identified morphologically as *S. ruber*/*S*. gr. *ruber*/*Scinax* sp. were resolved as *S. similis*, a widely distributed taxon, or as “*Scinax* sp. 27”, a sister lineage to *S. ruber* [122].

Similarly, DNA barcoding clarified the identity of *Adenomera* species: specimens initially classified as *Adenomera* sp. were resolved as *A. andreae*, *A. hylaedactyla*, and *A. kayapo.* The latter, a member of the *A. heyeri* group, was previously known from the Xingu-Tocantins interfluve, occurring north of the Tocantins River, northeast of Mato Grosso, and in Pará, specifically in the municipalities of Palestina do Pará (its type locality), Parauapebas (Carajás National Forest), and São Félix do Xingu [123]. Our study extends the knowledge distribution of *A. kayapo*, recording it in southeastern Pará, specifically in the municipalities of Conceição do Araguaia, Marabá, Ourilândia do Norte, and São Geraldo do Araguaia.

In addition to resolving taxonomic uncertainties, generating a DNA barcode library is crucial for future monitoring and conservation [124,125]. Traditional biodiversity surveys remain valuable, but integrating genetic data enhances the accuracy and sensitivity of species identification [11,14]. DNA barcoding, which relies on standardized DNA regions, has proven effective for documenting known biodiversity and detecting cryptic species. It enables reliable identification even in cases where morphology is inconclusive, such as juveniles, sterile samples, mixed collections, or degraded material. The reliability of these data depends on associating sequences with voucher specimens and comprehensive metadata to ensure reproducibility.

Despite its importance, genetic references for South American amphibians and squamate reptiles remain scarce. Recent initiatives have begun addressing this gap: for example, Koroiva et al. [11] created an amphibian barcode database from Mato Grosso do Sul, and Moraes et al. [126] combined morphological and COI barcoding for Amazonian herpetofauna. Our study complements these efforts by establishing a reference library for southeastern Pará, focusing on predominantly Amazonian species. This dataset expands available resources for taxonomy, monitoring, and conservation in a biodiversity-rich but understudied region.

Molecular markers such as COI and 16S rRNA are widely used for species delimitation in amphibians and reptiles (e.g., [11–18]). COI is the standard marker due to its high mutation rate, while 16S rRNA often provides greater resolution in amphibians [10,12]. In our analyses, 16S rRNA showed slightly better ASAP performance for amphibians, whereas COI was more effective for reptiles, highlighting the value of using complementary markers to ensure robust lineage delimitation.

According to the IUCN, 87.9% of the species recorded in southeastern Pará are classified as Least Concern (LC), 1.1% as Data Deficient (DD), and 11.0% have not yet been evaluated. The areas adjacent to the Carajás Mosaic are heavily anthropized and contain numerous forest fragments with low ecological connectivity. Nevertheless, they are critical as they serve as migration corridors, linking protected areas, including the Carajás Mosaic and surrounding conservation units. As highlighted by Castro et al. [112], these transitional zones should be considered in the development of regional conservation plans.

## Conclusion

Understanding the diversity of amphibians and squamate reptiles, together with generating genetic references, is crucial for conserving biodiversity in regions of high diversity and endemism such as the Amazon. Our study addressed significant knowledge gaps in underexplored areas, particularly within *canga* environments and associated forests, making an innovative contribution to taxonomy and systematics. The information generated here provides a basis for conservation and management strategies, especially for cryptic species, and establishes a curated DNA barcode library to support future monitoring and species delimitation studies.

Our findings also show that the effectiveness of molecular markers varies across taxonomic groups. COI performed better for reptiles, while 16S rRNA was slightly superior for amphibians, underscoring the importance of marker choice according to study objectives. Combining both markers is ideal to maximize precision in species delimitation.

Spatial variation in species composition and the presence of exclusive species in each locality highlight the influence of geographic heterogeneity on community structure. In fragmented landscapes, such as Conceição do Araguaia, remnant habitats retain high conservation value by serving as ecological corridors between protected areas.

Overall, this study advances knowledge of Amazonian herpetofauna and provides a solid foundation for ecological monitoring, biogeographic research, and conservation planning in southeastern Pará, a region under increasing anthropogenic pressure and habitat loss.

## Supporting information

S1 Fig Majority-rule consensus tree based on maximum likelihood evidencing the phylogenetic relationships among amphibian species from the Serra dos Carajás using the mitochondrial genes COI and 16S rRNA. Bootstrap support values are indicated near clade branches.
(ZIP)
