# Supplementary material for: Unraveling the herpetofauna diversity in canga and forest ecosystems of the Eastern Amazon
Source: PLoS One. 2025 Nov 26;20(11):e0332753. doi: 10.1371/journal.pone.0332753 (PMC12654886; doi:10.1371/journal.pone.0332753)
Supplement: S1 Fig — Bootstrap support values are indicated near clade branches. (ZIP) [file pone.0332753.s001.zip › Supporting Information/S1_Table.docx]

**S1 Table.** **Details of primer sets used in this study.** Primer set selected for DNA amplification of amphibian and squamate reptile specimens examined during this research.

| **Primer** | **Sequence 5’–3’** |  | **Target** | **Reference** |
| --- | --- | --- | --- | --- |
| COI-ReptBCF | TCAACAAACCAYAAAGAYATYGG |  | Reptiles | [1] |
| COI-ReptBCR | TAAACTTCAGGGTGGCCRAARAATCA |  | Reptiles |  |
| 16Sar | CGCCTGTTTATCAAAAACAT |  | Universal | [2] |
| 16Sbr | CCGGTCTGAACTCAGATCACGT |  | Universal |  |

**Reference**

1. Castañeda MR, de Queiroz K. Phylogenetic relationships of the Dactyloa clade of *Anolis* lizards based on nuclear and mitochondrial DNA sequence data. Mol Phylogenet Evol. 2011;61(3):784–800. doi: 10.1016/j.ympev.2011.07.004
2. Palumbi SR, Martin AP, Romano S, McMillan WO, Stice L, Grabowski G. 1991. The simple fool's guide to PCR. University of Hawaii.
