# Supplementary material for: Unraveling the herpetofauna diversity in canga and forest ecosystems of the Eastern Amazon
Source: PLoS One. 2025 Nov 26;20(11):e0332753. doi: 10.1371/journal.pone.0332753 (PMC12654886; doi:10.1371/journal.pone.0332753)
Supplement: S1 Fig — Bootstrap support values are indicated near clade branches. (ZIP) [file pone.0332753.s001.zip › Supporting Information/S2_File.pdf]

Supporting Information: S2 Files

Unraveling the herpetofauna diversity in *canga* and forest ecosystems of the Eastern Amazon

Prudente et al.

This document includes:

S2 Fig 1. Advertisement call of *Adenomera saci*.
S2 Fig 2. Advertisement call of *Allobates carajas*.
S2 Fig 3. Advertisement call of *Dendropsophus anataliasiasi.*

Bioacoustic notes of selected species from Southeastern Pará.

Adenomera saci

On March 10, 2022, we collected an adult male specimen of *A. saci* (MPEG 44015) near Lagoa Coró-coró, approximately 29 km northwest of Conceição do Araguaia, Pará (-8.054694°, -49.425028°; 248 m asl). The specimen was found in a *canga* environment, hiding among grass in soaked soil, at the onset of the night (19:10 h). We also recorded the advertisement calls of this specimen, along with several other individuals in the same locality.

The calls were characterized by tonal notes emitted at a rate of 2.0–2.1 notes per second, with a note duration ranging from 160 to 193 ms (averaging 180.5 ± 9.0 ms; n = 11). The internote interval ranged from 286 to 465 ms (averaging 327.8 ± 47.6 ms; n= 11). The fundamental frequency varied between 1.83 and 1.92 kHz (averaging 1.98 ± 0.03 kHz; n = 11), while the dominant frequency, corresponding to the second harmonic, ranged from 3.65 to 3.85 kHz (averaging 3.79 ± 0.06 kHz; n = 11) (S2 Fig 1).

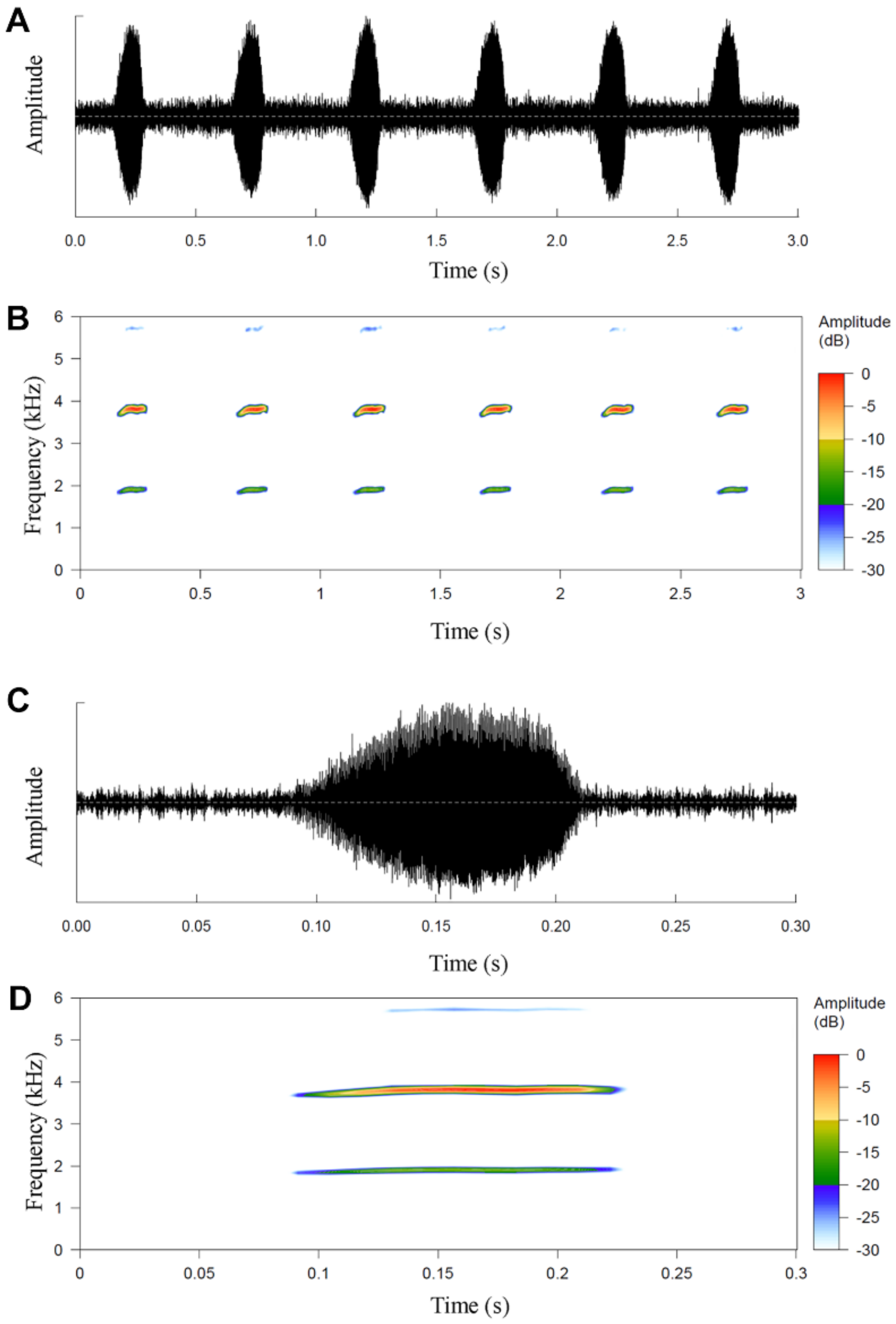

S2 Fig 1. Advertisement call of *Adenomera saci*. (A) Oscillogram and (B) spectrogram of a 3s fragment of a call. Detailed view of the (C) oscillogram and (D) spectrogram of a 0.3s section of the same recording, depicting a single note.

Allobates carajas

On December 5, 2021, we collected an adult female *Allobates* (MPEG 43944, SVL: 19.9 mm) at the southern foothill of Serra Arqueada, São Félix do Xingu, Pará (-6.515722°, -51.148778°; 372 m asl). The specimen was found active in the late morning (11:16 h), jumping on fallen palm fronds in an area characterized by soaked soil within a patch of palm forest along a small stream. During the collection, calls of several males were heard emanating from the forest floor. Although we were unable to capture additional specimens, we continued to hear and observe other individuals during the morning and evening (from 08:19 to 17:02 h) on December 5 and 7, 2021. On December 7, we recorded the call of a single male at 17:03 h.

The calls of a male we recorded were characterized by short trills consisting of 5–14 tonal notes, produced at a rate of 2.6–3.4 notes per second (n = 5 calls). Each note had a duration ranging from 34 to 53 ms (averaging 42.5 ± 6 ms; n = 24), with an inter-note interval spanning 237 to 562 ms (average 359.0 ± 88.6 ms; n = 24). The fundamental frequency ranged from 2.73 to 2.82 kHz (average 2.79 ± 0.02 kHz; n = 21), while the dominant frequency, corresponding to the second harmonic, ranged from 5.04 to 5.64 kHz (averaging 5.52 ± 0.13 kHz; n = 24) (S2 Fig 2).

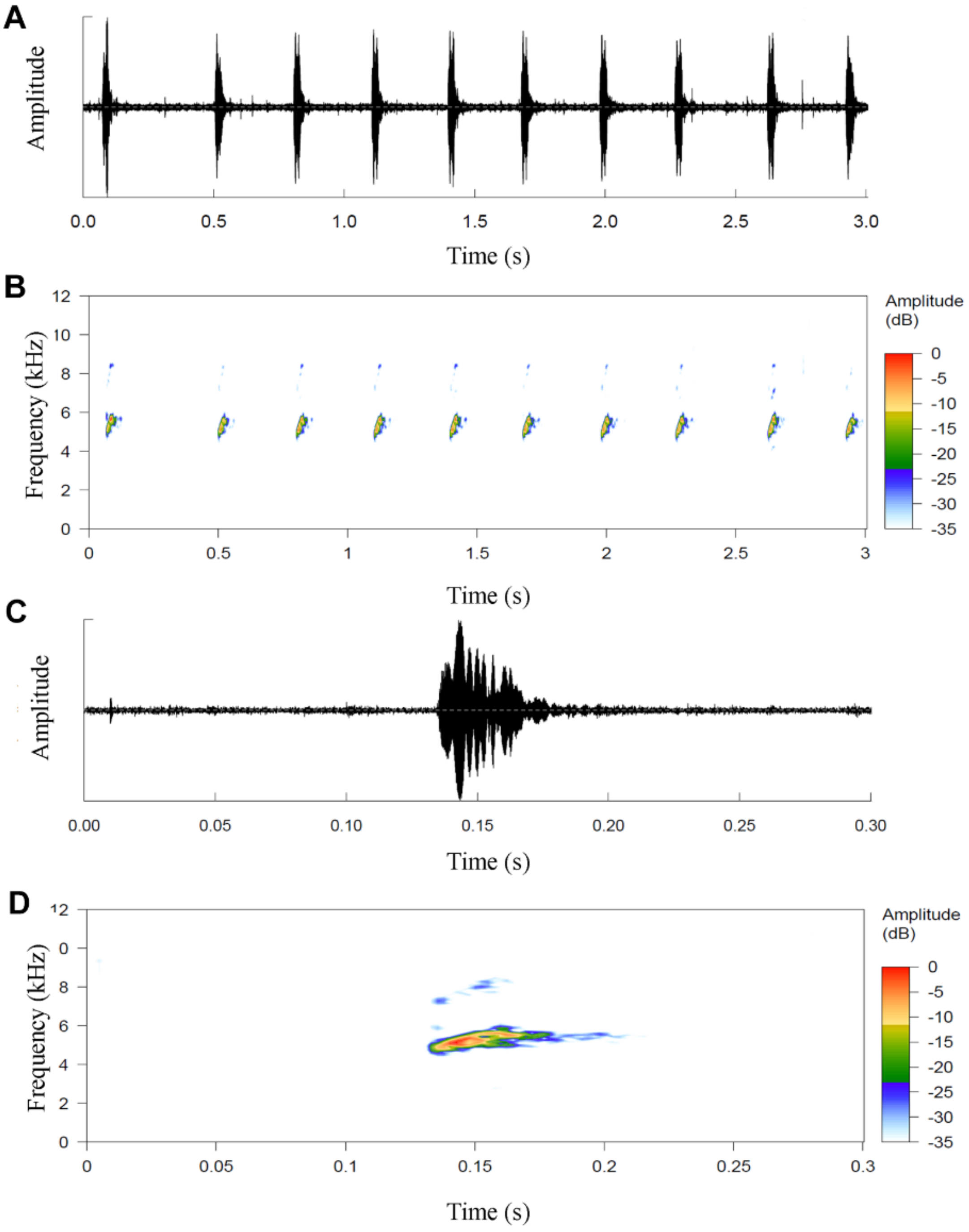

S2 Fig 2. Advertisement call of *Allobates carajas*. (A) Oscillogram and (B) spectrogram of a 3s fragment of a call. Detailed view of the (C) oscillogram and (B) spectrogram of a 0.3s long section of the same recording depicting a single note.

Dendropsophus anataliasiasi

Between March 10 and 11, 2022, we collected three adult males of *Dendropsophus anataliasiasi* in canga of Conceição do Araguaia, southeastern Pará. The first two specimens (MPEG 44051 and 44052, SVL: 23.4 and 19.7 mm, respectively) were found on March 10, 2022, calling from grasses (19:40 h) near a small temporary watercourse at Lagoa Coró-coró (-8.054694°, -49.425028°; 248 m asl). The third specimen (MPEG 44053, SVL: 22.6 mm) was found at 20:10 h on March 11, 2022, calling from a small bush near a temporary pond (-8.229944°, -49.779944°; 215 m asl). Vocalizations of this last specimen were recorded, showing two types of pulsed notes: note A (3–5 pulses, lasting 28–34 ms) and note B (1–3 pulses, lasting 14–19 ms), emitted in composite calls (A + B) with silent intervals of 125–157 ms between notes (S2 Fig 3).

The vocalizations are emitted as call series, which include an introductory simple call (represented by note A), followed by one or two composite calls of two, occasionally three, notes (A + B or A + B + B). Finally, the dominant frequency corresponds to the fundamental harmonic and ranges between 3.51–3.83 kHz (3.71 ± 0.07; n = 48); it does not differ between notes A and B. In addition to its particular arrangement of two type of notes in composite calls, the vocalization of *D. anataliasiasi* from Conceição do Araguaia agrees in most of its spectral and temporal attributes with that described by Teixeira and Giaretta (2015) for topotypes of *D. anataliasiasi* from Tocantins, except for its slightly fewer pulses in both A and B notes (A with 5–9 and B with 2–4 in topotypes from Tocantins) and its slightly lower dominant frequency (3.84–4.31 kHz in topotypes). We consider these differences minor and highlight that we have likely described only a fraction of the actual call variation of *D. anataliasiasi* from Conceição do Araguaia, as our bioacoustics characterization is based on call records from a single specimen.

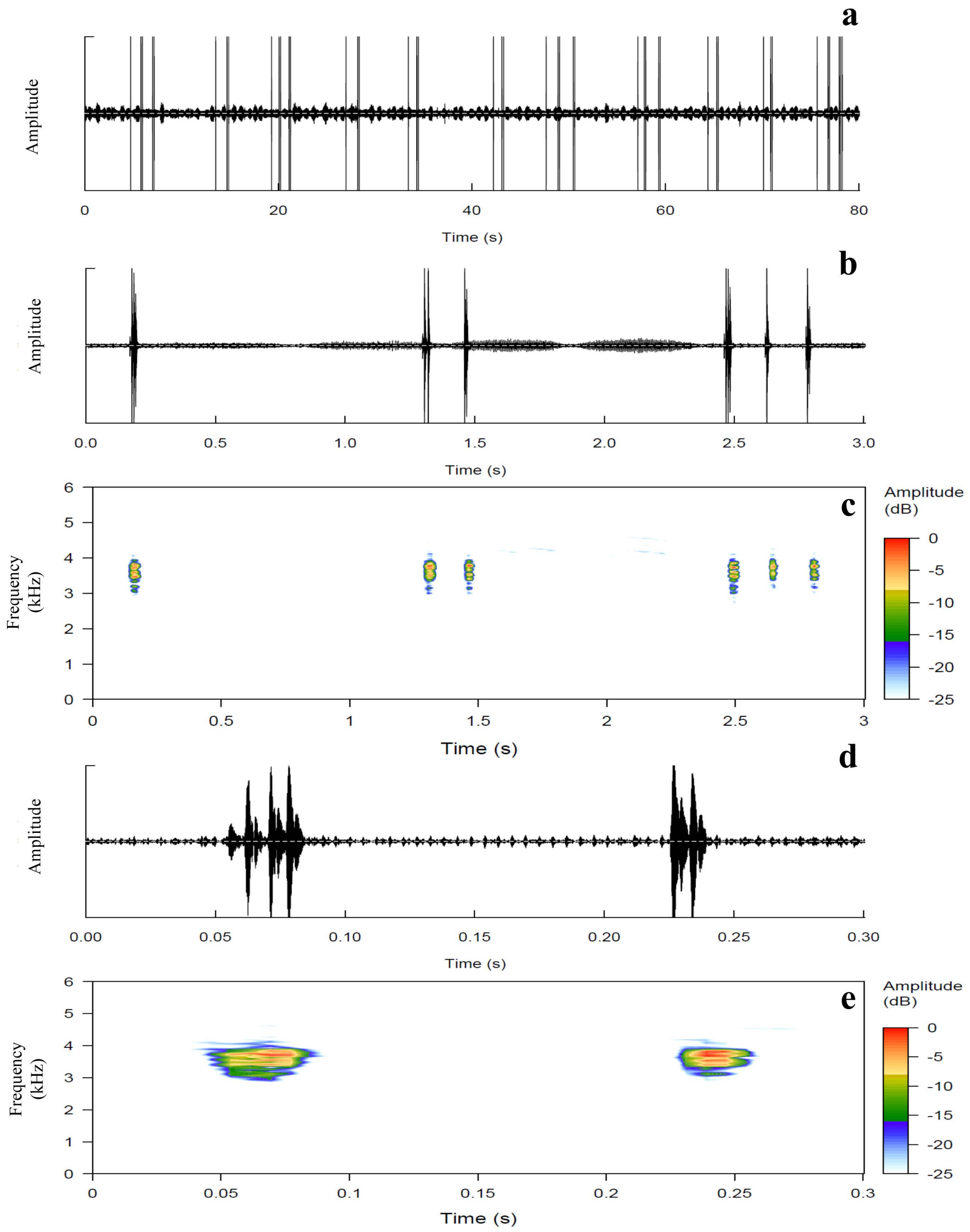

S2 Fig 3. Advertisement call of *Dendropsophus anataliasiasi.* (A) Oscillogram of an 80s fragment of call series. (B) Oscillogram and (C) spectrogram of a 3s call series exhibiting an introductory simple call represented by a note A, followed by two composite calls of two, and three notes (A + B and A + B + B, respectively). (D) Detailed view of the oscillogram and (E) spectrogram of a 0.3s section of the same recording, depicting a composite call containing a note A followed by a note B.
