# Supplementary material for: Unraveling the herpetofauna diversity in canga and forest ecosystems of the Eastern Amazon
Source: PLoS One. 2025 Nov 26;20(11):e0332753. doi: 10.1371/journal.pone.0332753 (PMC12654886; doi:10.1371/journal.pone.0332753)
Supplement: S1 Fig — Bootstrap support values are indicated near clade branches. (ZIP) [file pone.0332753.s001.zip › Supporting Information/S2_Table.docx]

**S2 Table. PCR conditions adopted for primer pairs selection**.

| **Primer pairs** | **Denaturation** | **Annealing and extension** | | | **Final extension** | **Hold** |
| --- | --- | --- | --- | --- | --- | --- |
| COI-ReptBCF / COI-ReptBCR | 95°C, 5m | 35 cycles | | | 72°C, 5m | 4°C |
|  |  | 95°C, 30s | 48°C, 30s | 72°C, 45s |  |  |
| 16Sar / 16Sbr | 95°C, 5m | 35 cycles | | | 72°C, 10m | 4°C |
|  |  | 94°C, 1m | 45°C, 1m | 72°C, 1m |  |  |
