# Supplementary material for: Unraveling the herpetofauna diversity in canga and forest ecosystems of the Eastern Amazon
Source: PLoS One. 2025 Nov 26;20(11):e0332753. doi: 10.1371/journal.pone.0332753 (PMC12654886; doi:10.1371/journal.pone.0332753)
Supplement: S1 Fig — Bootstrap support values are indicated near clade branches. (ZIP) [file pone.0332753.s001.zip › Supporting Information/S3_Table.pdf]

S3 Table. Species of amphibians recorded in southeastern Pará, Brazil, Eastern Amazon.

Taxon	Occurrence Areas				Habitat	IUCN
	CA	CM	ON/SX	SA		
ANURA						
**Allophrynidae** Savage, 1973						
Allophryne ruthveni Gaige, 1926		2,3			F,O,Pa	LC
**Aromobatidae** Grant et al., 2006						
Allobates carajas Simões *et al*., 2019		2,3	1,2	2	F	LC
Allobates crombiei (Morales, 2002)		2			F	LC
Allobates femoralis (Boulenger, 1884)		2,3	2		F,O	LC
**Bufonidae** Gray, 1825						
Amazophrynella cf. *xinguensis* Rojas-Zamora *et al*., 2018		2,3			F	-
Atelopus sp.		2,3			F	LC
Rhaebo guttatus (Schneider, 1799)	1	2,3	2	1,2	F,O,Pa	LC
Rhinella castaneotica (Caldwell, 1991)		2	2		F	LC
Rhinella dapsilis (Myers & Carvalho, 1945)		2	2	1,2	F,O	LC
Rhinella diptycha (Cope, 1862)		3	1,3	1,3	F,O	LC
Rhinella gr. *margaritifera* (Laurenti, 1768)		2,3	2	1,2	F,O,Pa	-
Rhinella mirandaribeiroi (Gallardo, 1965)	1	2,3	2	1,2	F,O,Pa	LC
**Centrolenidae** Taylor, 1951						
Hyalinobatrachium cf. *muiraquitan* Oliveira & Hernández-Ruz, 2017		2,3			F	-
Hyalinobatrachium iaspidiense (Ayarzagüena, 1992)		2			F	LC
**Ceratophryidae** Tschudi, 183						
Ceratophrys cornuta (Linnaeus, 1758)		2,3			F,O	LC
**Dendrobatidae** Cope, 1865 (1850)						
Adelphobates galactonotus (Steindachner, 1864)		2,3	2	1,2	F,O,Pa	LC
Ameerega flavopicta (Lutz, 1925)		2,3	1,2	1,2	F,O,Pa	LC
Ameerega hahneli (Boulenger, 1884)		3	2		F	LC
**Hylidae** Rafinesque, 1815						
Boana boans (Linnaeus, 1758)		2,3	2	1,2	F,O,Pa	LC
Boana cinerascens (Spix, 1824)		2,3	2		F,O,Pa	LC
Boana geographica (Spix, 1824)	1	2,3	2		F,O,Pa	LC
Boana multifasciata (Günther, 1859)	1	2,3	1,2	1	F,O,Pa	LC
Boana punctata (Schneider, 1799)	1	2,3		1,2	F,O,Pa	LC
Boana raniceps (Cope, 1862)	1	2,3		1,2	O,Pa	LC
Boana steinbachi (Boulenger, 1905)		2,3	2		O	LC
Dendropsophus aff. *minutus* (Peters, 1872)	1	3	1,3	1,2	F,O,Pa	-
Dendropsophus aff. *nanus* (Boulenger, 1889)	1	2			F,O,Pa	-
Dendropsophus aff. *triangulum* (Günther, 1869)		2,3	2	1	O,Pa	-
Dendropsophus anataliasiasi (Bokermann, 1972)	1				O	LC
Dendropsophus leucophyllatus (Beireis, 1783)		2,3	3		F	LC
Dendropsophus melanargyreus (Cope, 1887)	1	2,3	2	2	O,Pa	LC

Dendropsophus microcephalus (Cope, 1886)	1	2	2,3	2	O,Pa	LC
Dendropsophus minusculus (Rivero, 1971)		2,3			F,O	-
Dendropsophus minutus (Peters, 1872)		2,3	2,3		F,O,Pa	LC
Dendropsophus parviceps (Boulenger, 1882)		2,3			F,O	LC
Osteocephalus leprieurii (Duméril & Bibron, 1841)		2,3			Pa	LC
Osteocephalus oophagus Jungfer & Schiesari, 1995		2,3		1	O	LC
Osteocephalus taurinus Steindachner, 1862	1	2,3	2	1,2	F,O,Pa	LC
Pseudis tocantins Caramaschi & Cruz, 1998		2,3			Pa	LC
Scinax aff. c*ruentomma* (Duellman, 1972)		2,3			F,O,Pa	-
Scinax boesemani (Goin, 1966)		2,3	2,3		F,O,Pa	LC
Scinax fuscomarginatus (Lutz, 1925)	1	2,3			F,O,Pa	LC
Scinax garbei (Miranda-Ribeiro, 1926)		2,3			F,O	LC
Scinax aff. *ruber* (Laurenti, 1768)	1	2,3	1,2,3	1,2	F,O,Pa	-
Scinax nebulosus (Spix, 1824)	1	2,3	2,3	1,2	Pa	LC
Scinax similis (Cochran, 1952)	1,3	2,3	1,2,3		F,O	LC
Scinax x-signatus (Spix, 1824)		2,3	2	1	O	LC
Sphaenorhynchus lacteus (Daudin, 1800)		2,3			F,O,Pa	LC
Trachycephalus coriaceus (Peters, 1867)		2,3			F,Pa	LC
Trachycephalus resinifictrix (Goeldi, 1907)		2,3			F,O	LC
Trachycephalus typhonius (Linnaeus, 1758)	1	2,3	2	1	F,O,Pa	LC
**Leptodactylidae** Werner, 1896 (1838)						
Adenomera andreae (Müller, 1923)		2,3	1,2,3	2	F,O,Pa	LC
Adenomera hylaedactyla (Cope, 1868)		2	2,3	1,2	O,Pa	LC
Adenomera kayapo Carvalho et al., 2021	1	2,3	1,2	1,2	F Pa	LC
Adenomera saci Carvalho & Giaretta, 2013	1			2	O	LC
Engystomops freibergi (Donoso-Barros, 1969)		2,3	2		F,O,Pa	LC
Leptodactylus aff. *kilombo* Silva et al. 2020			2	1,2	O	-
Leptodactylus fuscus (Schneider, 1799)	1	2,3	2	1,2	F,O,Pa	LC
Leptodactylus gr. *melanonotus* (Hallowell, 1861)	1	2	2	1,2	F,O,Pa	-
Leptodactylus labyrinthicus (Spix, 1824)	1	2	1,2	2	F,O,Pa	LC
Leptodactylus leptodactyloides (Andersson, 1945)		2,3	2	1,2	F,O,Pa	LC
Leptodactylus macrosternum Miranda-Ribeiro, 1926	1	2,3		1,2	Pa	LC
Leptodactylus mystaceus (Spix, 1824)		2,3	2,3	1,2	F,O,Pa	LC
Leptodactylus paraensis Heyer, 2005		2,3	2,3	1	F,O,Pa	LC
Leptodactylus pentadactylus (Laurenti, 1768)		2,3	2,3	2	F,O,Pa	LC
Leptodactylus petersii (Steindachner, 1864)		2,3	1,2	1,2	F,O,Pa	LC
Leptodactylus podicipinus (Cope, 1862)	1		1		O	LC
Leptodactylus pustulatus (Peters, 1870)	1	3		1,2	Pa	LC
Leptodactylus rhodomystax Boulenger, 1884		2,3	2,3		F,O	LC
Leptodactylus syphax Bokermann, 1969		2,3		1,2	O	LC
Leptodactylus vastus Lutz, 1930	1		1	1,2	O	LC
Lithodytes lineatus (Schneider, 1799)		2,3			F,O	LC
Physalaemus centralis Bokermann, 1962	1				O	LC
Physalaemus aff. *cuvieri* Fitzinger, 1826	1	2,3	1,2	1,2	F,O,Pa	LC

Pseudopaludicola canga Giaretta & Kokubum, 2003	1,3	2,3		1,2,3	O	LC
Pseudopaludicola javae Silva et al. 2023	1				O	-
Pseudopaludicola mystacalis (Cope, 1887)		2		1	O,Pa	LC
**Microhylidae** Günther, 1858 (1843)						
Chiasmocleis avilapiresae Peloso & Sturaro, 2008		2,3			F,O,Pa	LC
Chiasmocleis centralis Bokermann, 1952	1				O	DD
Ctenophryne geayi Mocquard, 1904		2,3			F,O	LC
Elachistocleis magna Toledo, 2010	1	2,3	2	2	O	LC
**Odontophrynidae** Lynch, 1969						
Proceratophrys concavitympanum Giaretta et al., 2000		2,3	2	2	F,O,Pa	-
Proceratophrys cf. *cristiceps* (Müller, 1883)		2	1,2	1,2	F	-
**Phyllomedusidae** Günther, 1858						
Phyllomedusa bicolor (Boddaert, 1772)		2,3	2		F,O	LC
Phyllomedusa vaillantii Boulenger, 1882		2,3			F,O	LC
Pithecopus araguaius Haga, Andrade, Bruschi, Recco-Pimentel & Giaretta, 2017	1	3	1	1	O	LC
Pithecopus hypochondrialis (Daudin, 1800)		2,3	2	2	F,O,Pa	LC
**Pipidae** Gray, 1825						
Pipa arrabali Izecksohn, 1976		2,3	3		F,O	LC
Pipa pipa (Linnaeus, 1758)		2,3			F,O	LC
**Strabomantidae** Hedges et al., 2008						
Barycholos ternetzi (Miranda-Ribeiro, 1937)	1				F,O	LC
Pristimantis giorgii Oliveira et al., 2020	1	2,3	1,2	1,2	F,O,Pa	LC
Pristimantis latro Oliveira et al., 2017		2,3			F,O,Pa	LC
Pristimantis moa Oliveira et al., 2020				1	F	LC
GYMNOPHIONA						
**Caeciliidae** Rafinesque, 1814						
Caecilia tentaculata Linnaeus, 1758		2,3	3		F,O	LC
Caecilia gracilis Shaw, 1802		2,3			F,O,Pa	LC
**Siphonopidae** Bonaparte, 1850						
Brasilotyphlus guarantanus Maciel et al., 2009		2,3			F,O	LC
Microcaecilia sp.		2,3	1		F,O	-
Siphonops annulatus (Mikan, 1820)		2,3			F,O	LC
**Typhlonectidae** Taylor, 1968						
Potomotyphlus kaupii (Berthold, 1859)		2,3			F,O,Pa	LC

Abbreviations: CA: Conceição do Araguaia, CM: Carajás Mosaic, ON/SX: Ourilândia do Norte/São Félix do Xingu, SA: São Geraldo do Araguaia. Record source: Fieldwork= 1; Collection data (MPEG)= 2; Literature data= 3. Habitat: F= Forest, O= Open vegetation, O*= preferentially open areas, and occasionally forested environments; Pa= perianthropic. Information on the conservation status of the species was obtained from the IUCN Red List (https://www.iucnredlist.org), considering the following categories: Data Deficient (DD) and Least Concern (LC).

Literature used:

Araújo-Vieira K, Lourenço ACC, Lacerda JVA, Lyra ML, Blotto BL, Ron SR, et al. Treefrog diversity in the Neotropics: phylogenetic relationships of Scinaxini (Anura: Hylidae: Hylinae). S Am J Herpetol. 2023;27. https://doi.org/10.2994/SAJH-D-22-00038.1

Bernardo PH, Guerra-Fuentes RA, Matiazzi W, Zaher H. Checklist of amphibians and reptiles of Reserva Biológica do Tapirapé, Pará, Brazil. Check List. 2012;8(5):839–46.

Carvalho TR, Moraes LJC, Lima AP, Fouquet A, Peloso PLV, Pavan D, et al. Systematics and historical biogeography of Neotropical foam-nesting frogs of the *Adenomera heyeri* clade (Leptodactylidae), with the description of six new Amazonian species. Zool J Linnean Soc. 2021;191(2):395-433. https://doi.org/10.1093/zoolinnean/zlaa051

Cassundé GF, Sturaro ML, Maciel A, Prudente ALC, Sarmento JFM, Peloso P. The amphibians of Pará, Brazil. Bol Mus Para Emílio Goeldi Cienc Nat. 2022;17(2):445–73. https://doi.org/10.46357/bcnaturais.v17i2.782

Guayasamin JM, Cisneros-Heredia DF, McDiarmid RW, Peña P, Hutter CR. Glassfrogs of Ecuador: Diversity, Evolution, and Conservation. Diversity. 2020;12(6):222. https://doi.org/10.3390/d12060222

Haga IA, Andrade FS, Bruschi DP, Recco-Pimentel SM, Giaretta AA. Unrevealing the leaf frogs Cerrado diversity: a new species of *Pithecopus* (Anura, Arboranae, Phyllomedusidae) from Mato Grosso state, Brazil. PLoS One. 2017;12(9):e0184631. https://doi.org/10.1371/journal.pone.0184631

Mângia S, Koroiva R, Nunes PMS, Roberto IJ, Ávila RW, Sant'Anna C, Santana DJ, Garda AA. A new species of *Proceratophrys* (Amphibia: Anura: Odontophrynidae) from the Araripe Plateau, Ceará State, Northeastern Brazil. Herpetologica. 2018;74(3):255-268.

Martins IA, Jim J. Advertisement call of *Hyla jimi* and *Hyla elianeae* (Anura, Hylidae) in Botucatu, São Paulo, Brazil. Braz J Biol. 2004;64(3B):645–54. https://doi.org/10.1590/S1519-69842004000400012

Neckel-Oliveira S, Galatti U, Gordo M, Pinheiro LC, Maschio GF. Anfíbios de Carajás. In: Martins FD, Castilho AF, Campos J, Hatano FM, editores. Fauna da Floresta Nacional de Carajás: estudos sobre vertebrados terrestres. Belo Horizonte: Rona; 2012. p. 66–82.

Pereyra MO, Blotto BL, Baldo D, Chaparro JC, Ron SR, Elias-Costa AJ, et al. Evolution in the genus *Rhinella*: A total evidence phylogenetic analysis of Neotropical True Toads (Anura: Bufonidae). Bull Am Mus Nat Hist. 2021;447:1-156. https://doi.org/10.1206/0003-0090.447.1.1

Pinheiro LC, Bitar YOC, Galatti U, Neckel-Oliveira S, Santos-Costa MC. Amphibians from the southeastern state of Pará: Carajás Region, northern Brazil. Check List. 2012;8(4):693–702. https://doi.org/10.15560/8.4.693

Señaris JC, Ayarzagüena J. Revisión taxonómica de la familia Centrolenidae (Amphibia: Anura) de Venezuela. Sevilla: Publicaciones del Comité Español del Programa Hombre y Biosfera – Red IberoMaB de la UNESCO; 2005. 337 p.

Zina J, Silva GR, Loebmann D, Orrico VGD. The recognition of *Dendropsophus minusculus* (Rivero, 1971) (Hylidae, Dendropsophini) as a highly polymorphic, multi-domain distributed species. Braz J Biol. 2014;74(3):146-153. https://doi.org/10.1590/1519-6984.22912
