# Supplementary material for: Unraveling the herpetofauna diversity in canga and forest ecosystems of the Eastern Amazon
Source: PLoS One. 2025 Nov 26;20(11):e0332753. doi: 10.1371/journal.pone.0332753 (PMC12654886; doi:10.1371/journal.pone.0332753)
Supplement: S1 Fig — Bootstrap support values are indicated near clade branches. (ZIP) [file pone.0332753.s001.zip › Supporting Information/S4_Table.pdf]

S4 Table. Species of squamate reptiles recorded in southeastern Pará, Brazil, Eastern Amazon.

Taxon	Occurrence Areas				Habitat	IUCN
	CA	CM	ON/SX	SA		
AMPHISBAENIA Gray, 1844						
**Amphisbaenidae** Gray, 1825						
Amphisbaena alba Linnaeus, 1758		2,3		1	F,O	LC
Amphisbaena anomala (Barbour, 1914)		2		1	F	LC
Amphisbaena brasiliana (Gray, 1865)		2,3			F	LC
Amphisbaena fuliginosa amazonica Vanzolini, 1951		2,3	3		F,O	LC
Amphisbaena miringoera Vanzolini, 1971		3			?	LC
Amphisbaena mitchelli Procter, 1923		2,3			F,O	LC
Leposternon microcephalum Wagler in Spix, 1824		2			?	LC
"LIZARDS"						
**Alopoglossidae** Goicoechea et al., 2016						
Alopoglossus angulatus (Linnaeus, 1758)		2,3			F	LC
**Anolidae** Cocteau, 1836						
Dactyloa punctata (Daudin, 1802)		2,3	2	2	F	LC
Norops brasiliensis (Vanzolini & Williams, 1970)	1,3	2,3	1	2	O	LC
Norops fuscoauratus (D'Orbigny, 1837)		2,3	2,3	2	F	LC
Norops ortonii (Cope, 1868)		2,3	3		F	LC
**Gekkonidae** Oppel, 1811						
Hemidactylus mabouia (Moreau de Jonnès, 1818)		2,3	2,3	1,2	Pa	LC
**Gymnophthalmidae** Fitzinger, 1826						
Arthrosaura kockii (Lidth de Jeude, 1904)		2,3	2		F	LC
Arthrosaura reticulata (O'Shaughnessy, 1881)		2,3	2		F	LC
Bachia flavescens (Bonnaterre, 1789)		2,3			F	LC
Cercosaura argulus Peters, 1862		2,3	2		F	LC
Cercosaura eigenmanni (Griffin, 1917)			3		F	LC
Cercosaura ocellata Wagler, 1830		2,3	2,3		F	LC
Cercosaura olivacea (Gray, 1845)				1,2,3	O	-
Colobosaura modesta (Reinhardt & Luetken, 1862)	1,3	2,3	3	2	F,O	LC
Micrablepharus atticolus Rodrigues, 1996	1,3			2,3	F,O	LC
Neusticurus bicarinatus (Linnaeus, 1758)		2,3			F	LC
Potamites aff. *ecpleopus* (Cope, 1875)		2,3	1,2	1,3	F	-
Rhachisaurus brachylepis (Dixon, 1974)		2,3			F	DD
Tretioscincus agilis (Ruthven, 1916)		2,3	2		F	LC
**Hoplocercidae** Frost & Etheridge, 1989						
Hoplocercus spinosus Fitzinger, 1843		2,3			F,O	LC
**Iguanidae** Gray, 1827						
Iguana iguana iguana (Linnaeus, 1758)		2,3		2	F,O	LC
**Leiosauridae** Frost, Etheridge, Janies & Titus, 2001						
Enyalius leechii (Boulenger, 1885)		2,3			F	LC
**Phyllodactylidae** Gamble et al., 2008						
Gymnodactylus amarali Barbour, 1925				1,2,3	O	LC

Phyllopezus aff. *pollicaris* (Spix, 1825)				1,2,3	F,O,Pa	LC
Thecadactylus rapicauda (Houttuyn, 1782)		2,3	1,2	1,3	F	LC
**Polychrotidae** Fitzinger, 1843						
Polychrus acutirostris Spix, 1825		2,3		2,3	F,O	LC
Polychrus marmoratus (Linnaeus, 1758)		2,3	1,3	1	F,O,Pa	LC
**Scincidae** Oppel, 1811						
Copeoglossum nigropunctatum (Spix, 1825)	3	2,3	2	1,2	F,O	LC
Notomabuya frenata (Cope, 1862)	1,3	2,3		2	F,O,Pa	LC
**Sphaerodactylidae** Underwood, 1954						
Chatogekko amazonicus (Andersson, 1918)		2,3	2,3		F	LC
Gonatodes eladioi Nascimento, Avila-Pires & Cunha, 1987		2,3	2,3		F	LC
Gonatodes humeralis (Guichenot, 1855)	1	2,3	1,2,3	1,2,3	F	LC
**Teiidae** Gray, 1827						
Ameiva ameiva (Linnaeus, 1758)	3	2,3	1,2	2,3	F,O	LC
Cnemidophorus cryptus Cole & Dessauer, 1993		2,3			O	LC
Cnemidophorus lemniscatus (Linnaeus, 1758)		3			O	LC
Kentropyx altamazonica (Cope, 1876)		3			F,O,Pa	LC
Kentropyx calcarata Spix, 1825	3	2,3	1,2	1,2,3	F	LC
Salvator merianae Duméril & Bibron, 1839		2,3		3	F,O*	LC
Tupinambis teguixin (Linnaeus, 1758)	3	2,3			F	LC
**Tropiduridae** Bell in Darwin, 1843						
Plica plica (Linnaeus, 1758)		2,3	2		F	LC
Plica umbra ochrocollaris (Spix, 1825)		2,3	2,3		F	LC
Tropidurus oreadicus Rodrigues, 1987	1	2,3	1,3	1,2,3	O	LC
Uranoscodon superciliosus (Linnaeus, 1758)		2,3	1,2		F	LC
SERPENTES						
**Aniliidae** Stejneger, 1907						
Anilius scytale (Linnaeus, 1758)		2,3	2	2	F	LC
**Anomalepididae** Taylor, 1939						
Liotyphlops ternetzii (Boulenger, 1896)		2,3	2,3		F,O	LC
**Boidae** Gray, 1825						
Boa constrictor constrictor Linnaeus, 1758	3	2,3	2	3	F,O	LC
Corallus batesii (Gray, 1860)		2,3	3		F	LC
Corallus hortulana (Linnaeus, 1758)	2	2,3	1,2,3		F	LC
Epicrates cenchria (Linnaeus, 1758)		2,3			F	LC
Eunectes murinus (Linnaeus, 1758)		2,3	2,3	1	F,O	LC
**Colubridae** Oppel, 1811						
Chironius carinatus (Linnaeus, 1758)		2,3			F,O	LC
Chironius exoletus (Linnaeus, 1758)		2,3	2,3		F,O	LC
Chironius flavolineatus (Jan, 1863)		2,3		1	O	LC
Chironius fuscus (Linnaeus, 1758)		2,3	2,3		F	LC
Chironius multiventris Schmidt & Walker, 1943		2,3	3		F	LC
Chironius scurrulus (Wagler in Spix, 1824)		2,3	2		F	LC
Dendrophidion dendrophis (Schlegel, 1837)		2,3	2		F,O	LC
Drymarchon corais (Boie, 1827)	3	2,3	3	3	F,O	LC

Drymoluber dichrous (Peters, 1863)	3	2,3	2,3		F,O	LC
Leptophis ahaetulla (Linnaeus, 1758)		2,3	2		F	LC
Mastigodryas boddaerti boddaerti (Sentzen, 1796)		2,3	1,2	3	F	LC
Oxybelis aeneus (Wagler in Spix, 1824)		2,3	1	1	F,O	LC
Oxybelis fulgidus (Daudin, 1803)		2,3	2		F	LC
Palusophis bifossatus (Raddi, 1820)	3				F	LC
Phrynonax polylepis (Peters, 1867		2,3			F	LC
Rhinobothryum lentiginosum (Scopoli, 1785)		2,3			F	LC
Spilotes pullatus pullatus (Linnaeus, 1758)		2,3	2,3	1,3	F,O	LC
Spilotes sulphureus sulphureus (Wagler in Spix, 1824)	3	2,3	2	3	F,O	LC
Tantilla melanocephala (Linnaeus, 1758)		2,3	2,3		F	LC
**Dipsadidae** Bonaparte, 1838						
Adelphostigma quadriocellatus Santos-Jr et al., 2008		2,3			F	LC
Apostolepis nelsonjorgei De Lema & Renner, 2004	2,3	2			F	LC
Apostolepis nigrolineata (Peters, 1869)	2,3	2,3	3	2	F, O	LC
Atractus akerios Melo-Sampaio et al. 2021		2,3			F	-
Atractus albuquerquei Cunha & Nascimento, 1983		2,3	2,3		F	LC
Atractus latifrons (Günther, 1868)		2,3			F	LC
Atractus tartarus Passos, Prudente & Lynch, 2016		2,3	2,3		F	-
Chlorosoma viridissimum (Linnaeus, 1758)		2,3			F	LC
Clelia clelia (Daudin, 1803)		2,3	2		F, O	LC
*Clelia plumbe*a (Wied-Neuwied, 1820)		2,3			F	LC
Dipsas catesbyi (Sentzen, 1796)		2,3	3		F	LC
Dipsas indica indica Laurenti, 1768	3	2,3	3		F	LC
Dipsas mikanii septentrionalis (Cunha et al., 1980)		2		1,3	F, O	LC
Dipsas pavonina Schlegel, 1837		2,3	2,3		F	LC
Dipsas variegata (Duméril et al., 1854)		2,3			F	LC
Drepanoides anomalus (Jan, 1863)		2,3	1		F	LC
Dryophylax hypoconia 02 (Wagler, 1830)	1	2		1,2	O	LC
Erythrolamprus aesculapii aesculapii (Linnaeus, 1766)		2,3			F	LC
Erythrolamprus carajasensis (Cunha et al., 1985)		2,3			O	DD
Erythrolamprus miliaris miliaris (Linnaeus, 1758)		2			F	LC
Erythrolamprus oligolepis (Boulenger, 1905)		2,3	2		F	LC
Erythrolamprus poecilogyrus schotti (Schlegel, 1837)	1	2,3			F, O	LC
Erythrolamprus reginae (Linnaeus, 1758)		2	2	2,3	F	LC
Erythrolamprus taeniogaster (Jan, 1863)		3	2		F	LC
Erythrolamprus typhlus typhlus (Linnaeus, 1758)		2,3	3		F	LC
Helicops angulatus (Linnaeus, 1758)	3	2,3	2,3	2,3	F	LC
Helicops polylepis Gunther, 1861	3				F, O	LC
Helicops trivittatus (Gray, 1849)		2,3		2	F, O	LC
Hydrodynastes bicinctus (Herrmann, 1804)		2,3			F	LC
Hydrops martii (Wagler in Spix, 1824)		2,3			F	LC
Hydrops triangularis (Wagler in Spix, 1824)	3	2,3			F	LC
Imantodes cenchoa (Linnaeus, 1758)	3	2,3			F	LC
Leptodeira annulata annulata (Linnaeus, 1758)		2	2,3	1,2	F	LC

Oxyrhopus formosus (Wied, 1820)		2,3			F	LC
Oxyrhopus melanogenys melanogenys (Tschudi, 1845)		2,3	2	2,3	F, O	LC
Oxyrhopus petolarius digitalis (Reuss, 1834)		2,3	2,3		F	LC
Oxyrhopus trigeminus Duméril et al., 1854		2,3		1,2	O*	LC
Philodryas nattereri Steindachner, 1870				2,3	O	LC
Pseudoboa coronata Schneider, 1801		2,3	2,3		F, O	LC
Pseudoboa nigra (Duméril et al., 1854)		2,3	1	3	O*	LC
Pseudoeryx plicatilis plicatilis (Linnaeus, 1758)		2			F	LC
Sibon nebulatus (Linnaeus, 1758)		2,3			F	LC
Siphlophis cervinus (Laurenti, 1768)		2,3			F	LC
Siphlophis compressus (Daudin, 1803)		2,3			F, O	LC
Siphlophis worontzowi (Prado, 1940)		2,3			F	LC
Xenodon rabdocephalus rabdocephalus (Wied, 1824)		2,3			F, O	LC
Xenodon severus (Linnaeus, 1758)		2,3	2		F	LC
Xenopholis scalaris (Wucherer, 1861)		2,3			F	LC
Xenopholis undulatus (Jensen, 1900)		2,3			O	LC
Xenoxybelis argenteus (Daudin, 1803)		2,3			F	LC
**Elapidae** Boie, 1827						
Micrurus filiformis (Günther, 1859)		2,3			F, O	LC
Micrurus hemprichii (Jan, 1858)		2,3	2		F	LC
Micrurus lemniscatus (Linnaeus, 1758)		2,3			F, O	LC
Micrurus paraensis Cunha & Nascimento, 1973		2,3	3		F	LC
Micrurus spixii Wagler in Spix, 1824		2,3			F, O	LC
Micrurus surinamensis (Cuvier, 1817)		2,3			F	LC
**Leptotyphlopida**e Stejneger, 1892						
Siagonodon septemstriatus (Schneider, 1801)		2,3			F	LC
Trilepida fuliginosa (Passos et al., 2006)				2,3	F	LC
Trilepida macrolepis (Peters, 1857)		2,3			F	LC
**Typhlopidae** Gray, 1825						
Amerotyphlops reticulatus (Linnaeus, 1758)		2	2		F, O	LC
**Viperida**e Oppel, 1811						
Bothrops atrox (Linnaeus, 1758)	2,3	2,3	2,3	1	F	LC
Bothrops bilineatus bilineatus (Wied, 1821)		2,3			F	LC
Bothrops brazili Hoge, 1954		2,3			F	LC
Bothrops sp.	1				O	LC
Bothrops taeniatus Wagler in Spix, 1824		2,3			F	LC
Crotalus durissus Hoge, 1966				1,3	O*	LC
Lachesis muta (Linnaeus, 1766)		2,3	2,3		F	LC

Abbreviations: CA: Conceição do Araguaia, CM: Carajás Mosaic, ON/SX: Ourilândia do Norte/São Félix do Xingu, SA: São Geraldo do Araguaia. Record source: Fieldwork= 1; Collection data (MPEG)= 2; Literature data= 3. Habitat: F= Forest, O= Open vegetation, O*= preferentially open areas, and occasionally forested environments; Pa= perianthropic. Information on the conservation status of the species was obtained from the IUCN Red List (https://www.iucnredlist.org), considering the following categories: Data Deficient (DD) and Least Concern (LC).

Literature used:

Bernardo PH, Guerra-Fuentes RA, Matiazzi W, Zaher H. Checklist of amphibians and reptiles of Reserva Biológica do Tapirapé, Pará, Brazil. Check List. 2012;8(5):839-846

Cunha OR, Nascimento FP, Ávila-Pires TCS. Os répteis da área de Carajás, Pará, Brasil (Testudines e Squamata) I. Pub Avul Mus Para Emílio Goeldi. 1985;40:1-92.

Gamble T, Colli GR, Rodrigues MT, Werneck FP, Simons AM. Phylogeny and cryptic diversity in geckos (*Phyllopezus*; Phyllodactylidae; Gekkota) from South America's open biomes. Mol Phylogenet Evol. 2012;62:943–953. https://doi.org/10.1016/j.ympev.2011.11.033

Gomides SC, Teixeira Junior M, Leal F, Thomassen H, Cassimiro J, Sousa Recoder R, et al. Redescription and geographical distribution of a rare microteiid lizard: *Rhachisaurus brachylepis* (Squamata: Gymnophthalmidae). South Am J Herpetol. 2020;15:20–29. https://doi.org/10.2994/SAJH-D-17-00078.1

Mott T, Neto CSC, Filho KSC. *Amphisbaena miringoera* Vanzolini, 1971 (Squamata: Amphisbaenidae): new state record. Check List. 2011;7(5):594-595. https://doi.org/10.15560/7.5.594

Nascimento FP, Ávila-Pires TCS, Cunha OR. Os répteis da área de Carajás, Pará, Brasil (Testudines e Squamata) II. Pub Avul Mus Para Emílio Goeldi. 1987;3(1):33-65.

Nogueira CC, Argôlo AJS, Arzamendia V, Azevedo JA, Barbo FE, Bérnils RS, et al. Atlas of Brazilian snakes: verified point-locality maps to mitigate the Wallacean shortfall in a megadiverse snake fauna. South Am J Herpetol. 2019;14(sp1):1–274. https://doi.org/10.2994/SAJH-D-19-00120.1

Ribeiro-Júnior MA, Amaral S. Catalogue of distribution of lizards (Reptilia: Squamata) from the Brazilian Amazonia. IV. Alopoglossidae, Gymnophthalmidae. Zootaxa. 2017;4269(2):151-196. doi: 10.11646/zootaxa.4269.2.1. PMid:28610330.

Silva MB, Ribeiro-Júnior MA, Ávila-Pires TCS. A new species of *Tupinambis* Daudin, 1802 (Squamata: Teiidae) from Central South America. J Herpetol. 2018;52:94–110. https://doi.org/10.1670/16-036

Sturaro MJ, Rodrigues MT, Colli GR, Knowles LL, Avila-Pires TC. Integrative taxonomy of the lizards *Cercosaura ocellata* species complex (Reptilia: Gymnophthalmidae). Zool Anz. 2018;275:37–65. https://doi.org/10.1016/j.jcz.2018.04.004

Trevine VC, Grazziotin FG, Giraudo A; Sallesbery-Pinchera N, Vianna JA, Zaher H. The systematics of Tachymenini (Serpentes, Dipsadidae): An updated classification based on molecular and morphological evidence. Zool Scr. 2022;51:643–663. https://doi.org/10.1111/zsc.12565
