# Supplementary material for: Unraveling the herpetofauna diversity in canga and forest ecosystems of the Eastern Amazon
Source: PLoS One. 2025 Nov 26;20(11):e0332753. doi: 10.1371/journal.pone.0332753 (PMC12654886; doi:10.1371/journal.pone.0332753)
Supplement: S1 Fig — Bootstrap support values are indicated near clade branches. (ZIP) [file pone.0332753.s001.zip › Supporting Information/S5_File.pdf]

Supporting Information: S5 Files

Unraveling the herpetofauna diversity in *canga* and forest ecosystems of the Eastern Amazon

Prudente et al.

S5 Table 1. Sampling effort for amphibians across data sources and study areas. The table summarizes the approximate sampling effort for amphibians in each study area, including fieldwork (number of expeditions, sampling days and species analyzed), museum collections (number of vouchers available in collection and species analyzed), literature (number of previous surveys incorporated and species analyzed), and genetic sampling (number of individuals sequenced and species analyzed). Study areas: CA = Conceição do Araguaia, CM = Carajás Mosaic, ON/SX = Ourilândia do Norte/São Félix do Xingu, SA = São Geraldo do Araguaia.

Study Areas / Data Source	CA	CM	ON/SX	SA
**Fieldwork** (expeditions/sampling days/species analyzed)	2 exp. / 19 days / 36 spp.	0 exp. / 0 days / 0 spp.	2 exp. / 19 days / 18 spp.	2 exp. / 23 days / 40 spp.
**Museum specimens** (vouchers available/species analyzed)	0 vouchers / 0 spp.	>3500 vouchers / 84 spp.	>400 vouchers / 47 spp.	>200 vouchers / 39 spp.
**Literature** (previous surveys incorporated/ species analyzed)	1 survey / 2 spp.	6 surveys / 89 spp.	2 surveys / 17 spp.	1 survey / 2 spp.
**Genetic sampling** (individuals sequenced/species analyzed)	157 inds. / 33 spp.	27 inds. / 15 spp.	122 inds. / 29 spp.	78 inds. / 32 spp.

Zeros in fieldwork indicate no direct expeditions conducted during this study; species richness records for these areas derive exclusively from other data sources.
“Previous surveys” corresponds to published studies with species lists incorporated here.

S5 Table 2. Sampling effort for squamate reptiles across data sources and study areas. The table summarizes the approximate sampling effort for squamate reptiles in each study area, including fieldwork (number of expeditions, sampling days and species analyzed), museum collections (number of vouchers available in collection and species analyzed), literature (number of previous surveys incorporated and species analyzed), and genetic sampling (number of individuals sequenced and species analyzed). Study areas: CA = Conceição do Araguaia, CM = Carajás Mosaic, ON/SX = Ourilândia do Norte/São Félix do Xingu, SA = São Geraldo do Araguaia.

Study Areas / Data Source	CA	CM	ON/SX	SA
**Fieldwork** (expeditions/sampling days/species analyzed)	2 exp. / 19 days / 9 spp.	0 exp. / 0 days / 0 spp.	2 exp. / 19 days / 14 spp.	2 exp. / 23 days / 23 spp.
**Museum specimens** (vouchers available/species analyzed)	4 vouchers / 4 spp.	>3000 vouchers / 128 spp.	>150 vouchers / 52 spp.	>140 vouchers / 28 spp.
**Literature** (previous surveys incorporated/ species analyzed)	2 surveys / 21 spp.	6 surveys / 122 spp.	3 surveys / 38 spp.	2 surveys / 25 spp.
**Genetic sampling** (individuals sequenced/species analyzed)	30 inds. / 9 spp.	4 inds. / 4 spp.	47 inds. / 22 spp.	35 inds. / 24 spp.

Zeros in fieldwork indicate no direct expeditions conducted during this study; species richness records for these areas derive exclusively from other data sources.
“Previous surveys” corresponds to published studies with species lists incorporated here.
