# Supplementary material for: Unraveling the herpetofauna diversity in canga and forest ecosystems of the Eastern Amazon
Source: PLoS One. 2025 Nov 26;20(11):e0332753. doi: 10.1371/journal.pone.0332753 (PMC12654886; doi:10.1371/journal.pone.0332753)
Supplement: S1 Fig — Bootstrap support values are indicated near clade branches. (ZIP) [file pone.0332753.s001.zip › Supporting Information/S5_Table.docx]

**S3 Table. New records for barcodes generated in species examined in this study.** A list of species barcoded for the first time, compared to previous records available in the NCBI Database (accessed on Sep 14, 2024).

| **Amphibia** | **COI** | **16S rRNA** |
| --- | --- | --- |
| *Adenomera kayapo* |  | x |
| *Allobates carajas* | x |  |
| *Ameerega flavopicta* |  | x |
| *Hyalinobatrachium iaspidiense* | x |  |
| *Pithecopus araguaius* | x |  |
| *Pseudopaludicola canga* | x |  |
| *Pseudopaludicola javae* | x |  |
| **Squamate Reptiles** | **COI** | **16S rRNA** |
| *Apostolepis nigrolineata* | x | x |
| *Atractus albuquerquei* | x |  |
| *Cercosaura ocellata* | x |  |
| *Cercosaura olivacea* | x | x |
| *Chatogekko amazonicus* | x | x |
| *Colobosaura modesta* | x | x |
| *Copeoglossum nigropunctatum* | x | x |
| *Corallus hortulana* | x | x |
| *Drymoluber dichrous* | x | x |
| *Dryophylax hypoconia* 02 | x | x |
| *Erythrolamprus carajasensis* | x | x |
| *Eunectes murinus* | x | x |
| *Gonatodes eladioi* | x |  |
| *Gonatodes humeralis* | x |  |
| *Gymnodactylus amarali* | x |  |
| *Helicops angulatus* | x |  |
| *Kentropyx calcarata* | x |  |
| *Mastigodryas boddaerti* |  | x |
| *Micrablepharus atticolus* | x |  |
| *Norops brasiliensis* | x | x |
| *Notomabuya frenata* | x |  |
| *Oxyrhopus trigeminus* | x |  |
| *Phyllopezus* aff. *pollicaris* | x |  |
| *Pseudoboa nigra* | x |  |
| *Trilepida fuliginosa* | x |  |
| *Trilepida macrolepis* | x | x |
