# Supplementary figures and images for: Unraveling the herpetofauna diversity in canga and forest ecosystems of the Eastern Amazon
Source: PLoS One. 2025 Nov 26;20(11):e0332753. doi: 10.1371/journal.pone.0332753 (PMC12654886; doi:10.1371/journal.pone.0332753)

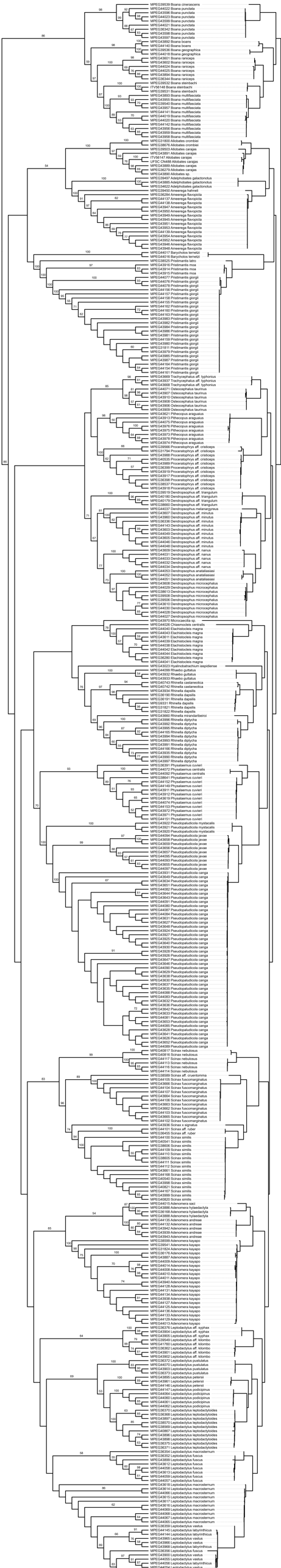

Supplement: S1 Fig — Bootstrap support values are indicated near clade branches. (ZIP) [file pone.0332753.s001.zip › Supporting Information/S1_Fig.pdf]

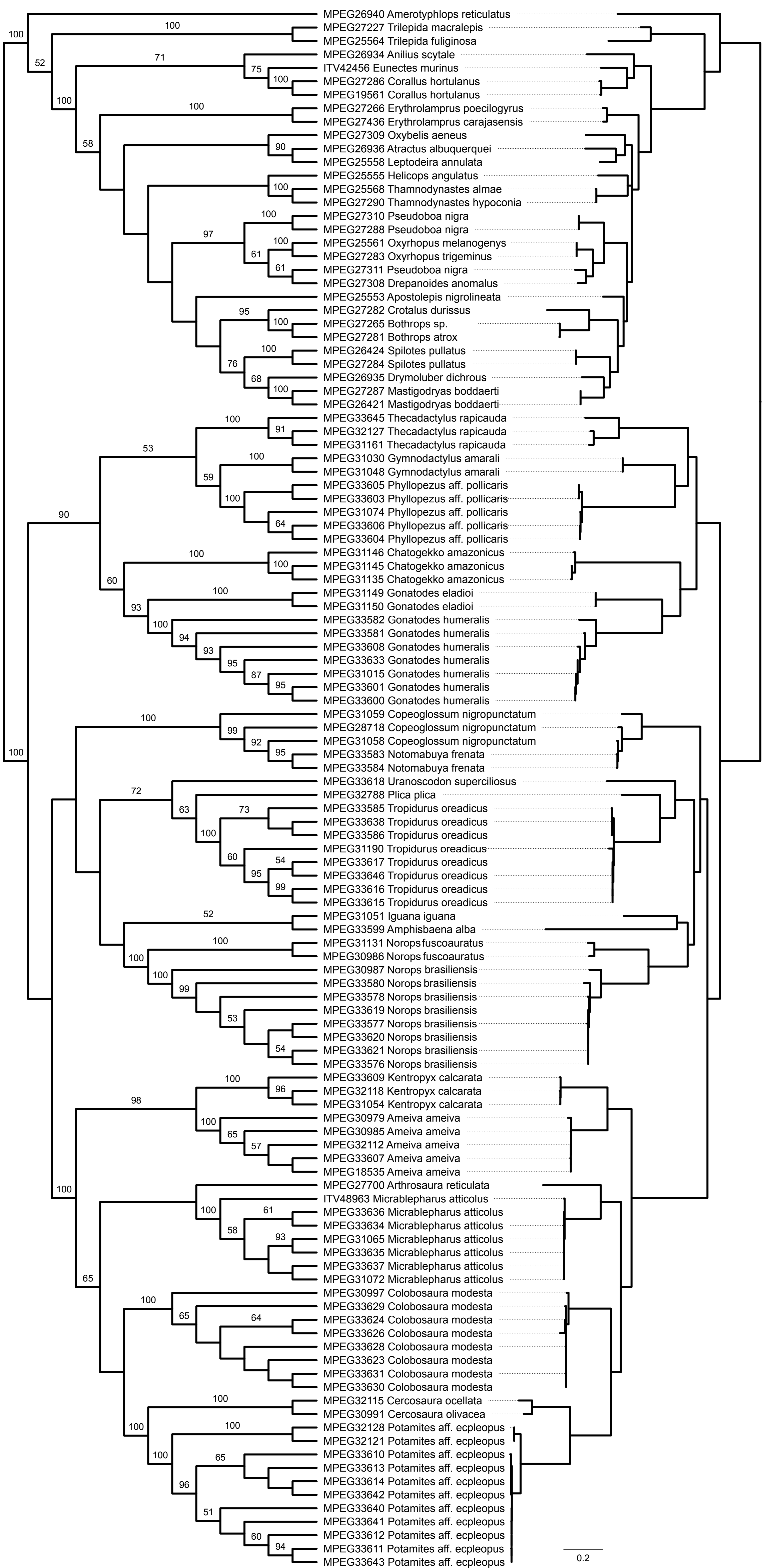

Supplement: S1 Fig — Bootstrap support values are indicated near clade branches. (ZIP) [file pone.0332753.s001.zip › Supporting Information/S2_Fig.pdf]

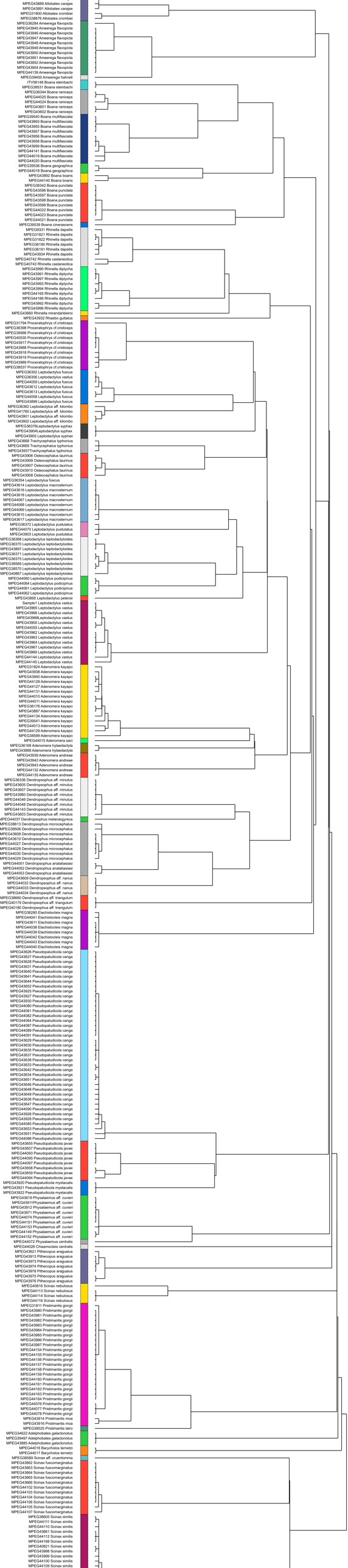

Supplement: S1 Fig — Bootstrap support values are indicated near clade branches. (ZIP) [file pone.0332753.s001.zip › Supporting Information/S3_Fig.pdf]

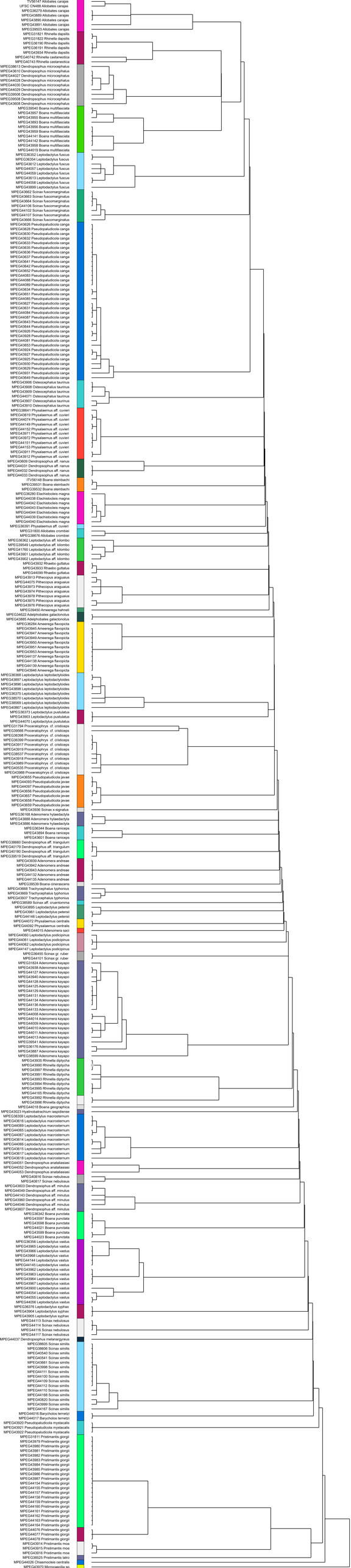

Supplement: S1 Fig — Bootstrap support values are indicated near clade branches. (ZIP) [file pone.0332753.s001.zip › Supporting Information/S4_Fig.pdf]

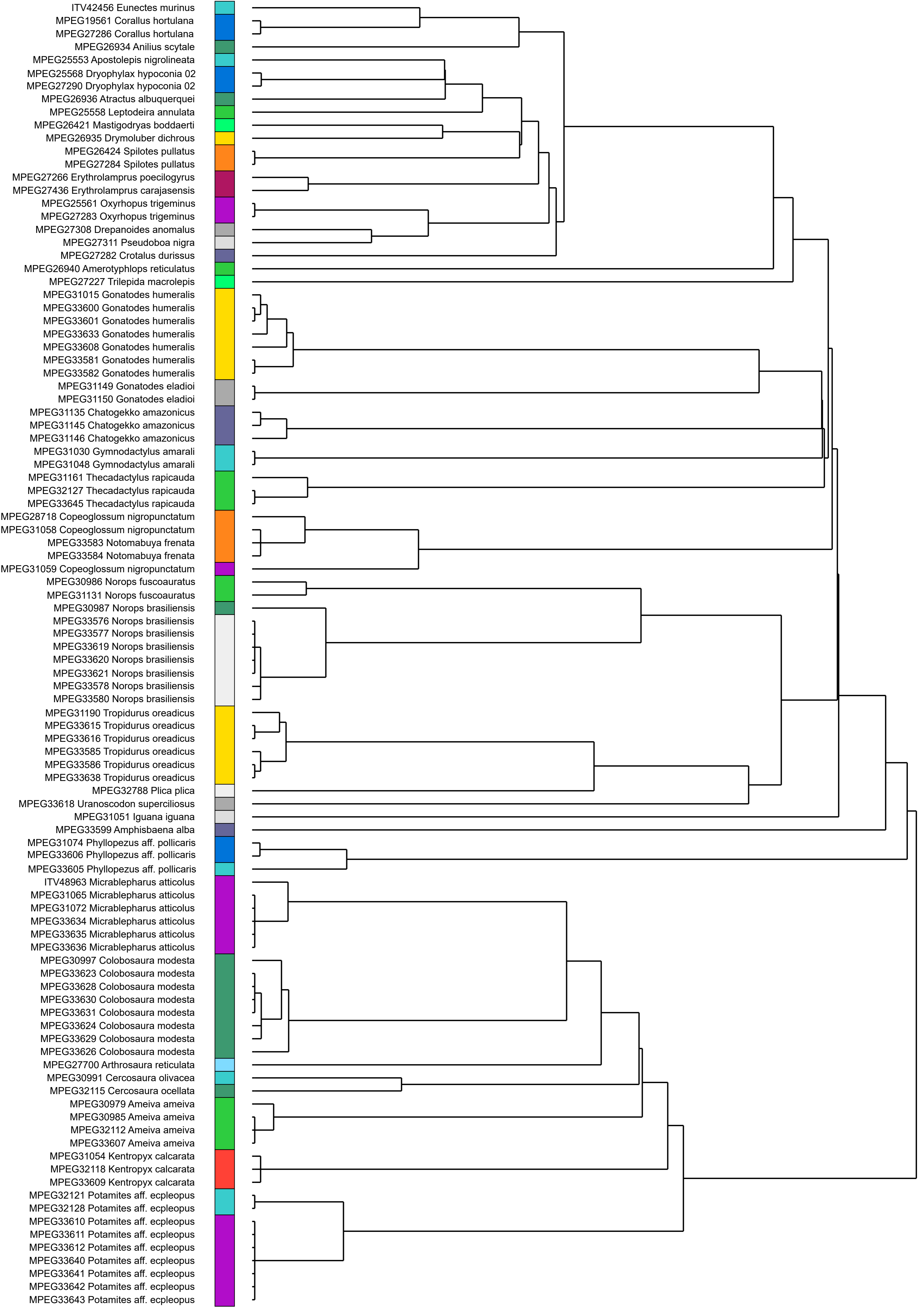

Supplement: S1 Fig — Bootstrap support values are indicated near clade branches. (ZIP) [file pone.0332753.s001.zip › Supporting Information/S5_Fig.pdf]

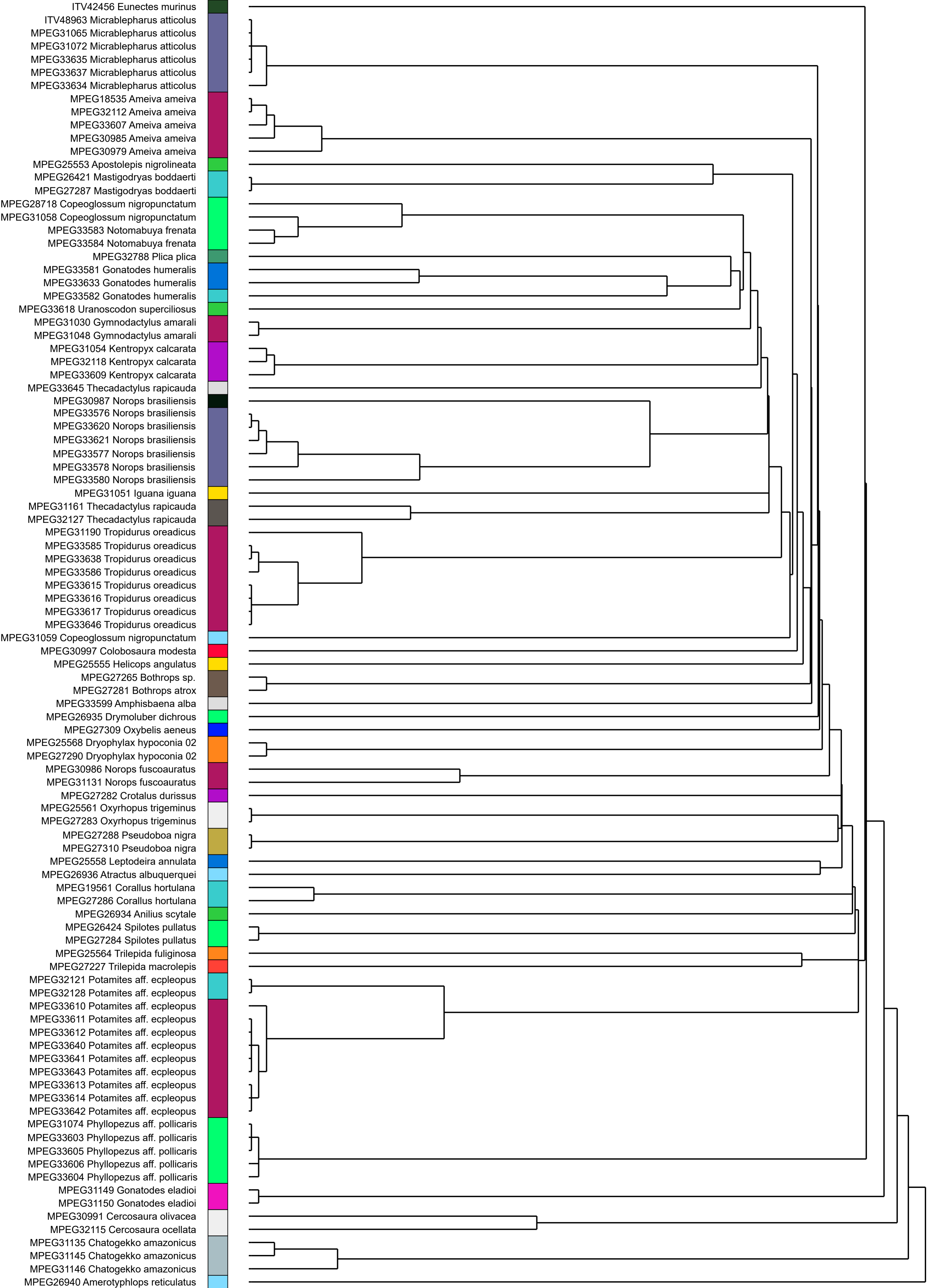

Supplement: S1 Fig — Bootstrap support values are indicated near clade branches. (ZIP) [file pone.0332753.s001.zip › Supporting Information/S6_Fig.pdf]
